# Phytochemical Shift from Condensed Tannins to Flavonoids in Transgenic *Betula pendula* Decreases Consumption and Growth but Improves Growth Efficiency of *Epirrita autumnata* Larvae

**DOI:** 10.1007/s10886-019-01134-9

**Published:** 2019-12-26

**Authors:** Paula Thitz, Lauri Mehtätalo, Panu Välimäki, Tendry Randriamanana, Mika Lännenpää, Ann E. Hagerman, Tommi Andersson, Riitta Julkunen-Tiitto, Tommi Nyman

**Affiliations:** 1 Department of Environmental and Biological Sciences, University of Eastern Finland, P.O. Box 111, FI-80101 Joensuu, Finland; 2 School of Computing, University of Eastern Finland, P.O. Box 111, FI-80101 Joensuu, Finland; 3 Ecology and Genetics Research Unit, University of Oulu, P.O. Box 8000, FI-90014 Oulu, Finland; 4 Present Address: Biocarelia Research Laboratory, FI-82580 Juurikka, Finland; 5 Department of Chemistry and Biochemistry, Miami University, Oxford, OH 45056 USA; 6 Kevo Subarctic Research Institute, Biodiversity Unit, University of Turku, FI-20014 Turku, Finland; 7 Present Address: Department of Ecosystems in the Barents Region, Norwegian Institute of Bioeconomy Research, NO-9925 Svanvik, Norway

**Keywords:** *Betula pendula*, Condensed tannins, *Epirrita autumnata*, Herbivory, Phenolics, RNA interference

## Abstract

Despite active research, antiherbivore activity of specific plant phenolics remains largely unresolved. We constructed silver birch (*Betula pendula*) lines with modified phenolic metabolism to study the effects of foliar flavonoids and condensed tannins on consumption and growth of larvae of a generalist herbivore, the autumnal moth (*Epirrita autumnata*). We conducted a feeding experiment using birch lines in which expression of dihydroflavonol reductase (*DFR*), anthocyanidin synthase (*ANS*) or anthocyanidin reductase (*ANR*) had been decreased by RNA interference. Modification-specific effects on plant phenolics, nutrients and phenotype, and on larval consumption and growth were analyzed using uni- and multivariate methods. Inhibiting *DFR* expression increased the concentration of flavonoids at the expense of condensed tannins, and silencing *DFR* and *ANR* decreased leaf and plant size. *E. autumnata* larvae consumed on average 82% less of DFRi plants than of unmodified controls, suggesting that flavonoids or glandular trichomes deter larval feeding. However, larval growth efficiency was highest on low-tannin DFRi plants, indicating that condensed tannins (or their monomers) are physiologically more harmful than non-tannin flavonoids for *E. autumnata* larvae. Our results show that genetic manipulation of the flavonoid pathway in plants can effectively be used to produce altered phenolic profiles required for elucidating the roles of low-molecular weight phenolics and condensed tannins in plant–herbivore relationships, and suggest that phenolic secondary metabolites participate in regulation of plant growth.

**Electronic supplementary material:**

The online version of this article (10.1007/s10886-019-01134-9) contains supplementary material, which is available to authorized users.

## Introduction

**Fig. 1:**
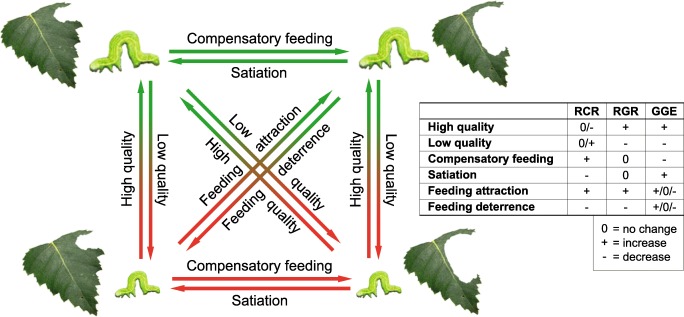
Interpretation of results when herbivore consumption and/or growth differ across alternative diets. For example, a low-quality diet decreases herbivore growth relative to their consumption (i.e., leads to low gross growth efficiency (GGE)). However, herbivores adapted to variable defenses of their hosts can use compensatory feeding to maintain their normal growth rate despite lowered GGE. Feeding deterrents in the diet decrease both herbivore consumption and growth, while changes in GGE depend on the relative magnitude of changes in RGR and RCR

Performance of herbivores on plants is determined by a complex interplay of factors including plant chemical defenses, morphological traits and general nutritional quality (Abdala-Roberts et al. 2016; Bedoya-Pérez et al. 2014; Couture et al. 2016;). Thus, for example leaf nitrogen, water and toughness are expected to interact with defensive compounds in determining consumption, growth and survival of folivores (Haukioja et al. 2002; Heroy et al. 2018; Scriber and Slansky 1981). Defensive compounds can act as feeding deterrents (i.e., limit consumption and, therefore, growth), antinutritive agents (i.e., decrease absorption of ingested nutrients), or as direct toxins. Both antinutritive compounds and toxins reduce gross growth efficiency (GGE, also known as efficiency of conversion of ingested food, ECI) of herbivores (Scriber and Slansky 1981). Interactive effects of nutrients and defensive compounds on insect performance are further modulated by insect behavioral responses such as compensatory feeding, in which an insect increases its consumption on a sub-optimal diet to meet its nutritional or energetic demands. Thus, inference of dietary quality requires simultaneous measurement of both herbivore consumption and growth, in order to separate physiological from behavioral effects (Fig. 1).

While the study of plant defenses against herbivorous insects has clear practical and theoretical importance, the interconnectedness of plant traits (Haukioja et al. 2002; Richards et al. 2016) poses a substantial challenge for identifying relevant compounds or traits. This problem is particularly pervasive in studies on genotypic or individual variation in plant susceptibility to herbivores (Stone et al. 2018), but also applies to experiments in which environmental treatments or gradients are used to modify potential defensive traits (Abdala-Roberts et al. 2016; Peltonen et al. 2010). Direct effects of individual phytochemicals on herbivores can be quantified from amended-diet experiments, in which artificial diets or detached leaves are supplemented with potential defensive chemicals (Ayres et al. 1997; Pandey et al. 2012). However, these studies do not take into account the synergistic effects of different phytochemicals (Richards et al. 2016), or their critical role in normal plant development (Jacobs and Rubery 1988).

Fortunately, developments in molecular biology now allow direct and targeted manipulation of the biosynthetic pathways that plants use for producing putative defensive compounds. Using genetically modified plants to investigate the effects of specific secondary metabolites on insect herbivores is a recent and potentially highly effective addition to the experimental toolbox of plant ecology (Brodeur-Campbell et al. 2006; Boeckler et al. 2014; Hjältén et al. 2013), and the approach has been successfully applied also for studying plant–pathogen interactions (Ullah et al. 2017). An especially versatile technique in this respect is stable RNA interference (RNAi), in which an artificially constructed sequence preceded by an active promoter is inserted into the genome of recipient cells (Mocellin and Provenzano 2004). The transferred sequence is designed so that it produces an RNA strand complementary to the mRNA produced by the targeted coding gene, thereby inhibiting its translation into proteins or enzymes (Mocellin and Provenzano 2004; Wesley et al. 2001).

Here, we applied stable RNAi to silence several key enzymes of the flavonoid pathway in silver birch (*Betula pendula* Roth), and then used the plants with altered phenolic secondary chemistry in experiments in which we measured growth and performance of larvae of a generalist herbivore, the autumnal moth (*Epirrita autumnata* Borkhausen). Silver birch is a pioneer species with high ecological and economic importance in Northern Eurasia (Hynynen et al. 2010; Zyryanova et al. 2010). The autumnal moth, in turn, is a common forest lepidopteran with a Holarctic distribution, causing outbreaks especially in the northern and mountainous parts of Fennoscandia (Jepsen et al. 2009; Tenow et al. 2007). Although the main host in outbreak populations is mountain birch (*B. pubescens* ssp. *pumila*), *E. autumnata* larvae are highly polyphagous and perform well on *B. pendula* (Haukioja et al. 1988).

Like other tree species, silver birch produces a wide variety of bioactive phenolics that help the plants to adapt and acclimate to their biotic and abiotic environment (Laitinen et al. 2000; Lattanzio et al. 2006). In *B. pendula*, antiherbivore properties have been suggested for many phenolics, including leaf and stem flavonoids and polymeric condensed tannins (Laitinen et al. 2004; Martemyanov et al. 2012; Mutikainen et al. 2000; Valkama et al. 2005a). Flavonol glycosides, a class of flavonoids, constitute a major proportion of low-molecular weight phenolics in *B. pendula* leaves, whereas non-flavonoid phenolic acids are normally present in small amounts (Laitinen et al. 2000). Leaf-surface phenolics, closely associated with glandular trichomes, include various flavonoid aglycones with highest concentrations in buds and very young leaves (Keinänen et al. 1999; Valkama et al. 2004).

**Fig. 2:**
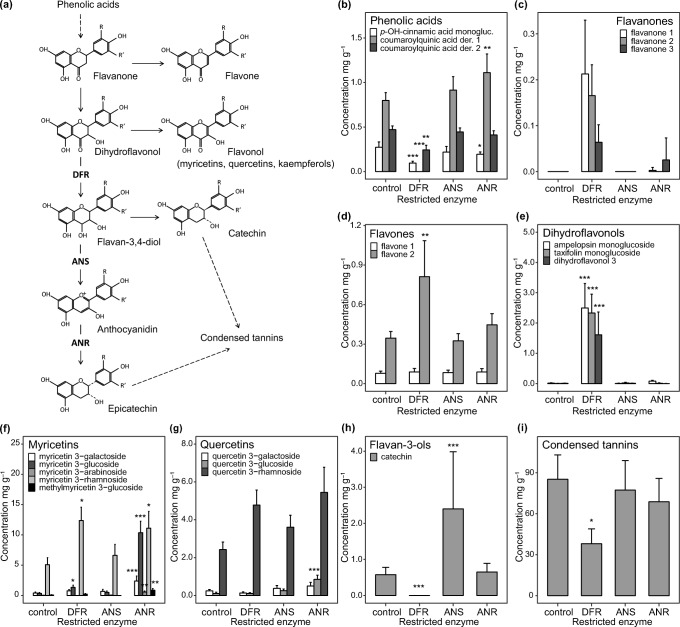
**(a)** The location of the three enzymes inhibited using RNA interference along the flavonoid pathway (DFR, dihydroflavonol reductase; ANS, anthocyanidin synthase; ANR, anthocyanidin reductase). **(b–i)** Concentrations of different phenolic compounds in leaves of the control and modified *Betula pendula* lines. Error bars show ±1 SE, asterisks denote statistically significant differences compared to the control line at 1/8 degrees of freedom in multi- or univariate linear mixed models at 0.01 < *P* < 0.05 (*), 0.001 < *P* < 0.01 (**), or *P* < 0.001 (***). Only flavanones and compounds present in the unmodified control line are shown. Note the different scales in the figures

As in other land plants, the biosynthetic pathway producing phenolics in *B. pendula* originates from phenylalanine, an aromatic amino acid (Cheynier et al. 2013), and proceeds via various phenolic acids to the production of flavonoids with their characteristic three-ringed structures (Fuglevand et al. 1996; Li et al. 1993). In the flavonoid pathway (Fig. 2a), this basic structure is further modified to yield, for example, flavonols, anthocyanidins and condensed tannins (Winkel-Shirley 2002a). Structural modification of compounds along the pathway is catalyzed by a succession of specific enzymes, rendering the process amenable to experimental manipulation by RNAi: restricting the activity of particular enzymes along the pathway should decrease allocation of photosynthates into products downstream of the inhibited enzyme, and thereby increase the levels of intermediate compounds and alternative products (Fig. 2a). This possibility is especially interesting in a species such as *B. pendula*, where antiherbivore functions have been suggested for both intermediate compounds and the various end products of the flavonoid pathway.

To answer the request of Barbehenn and Constabel (2011) for herbivory experiments using transgenic plants with reduced levels of condensed tannins, we used replicate *B. pendula* lines with suppressed expression of three key enzymes of the flavonoid pathway (Fig. 2a) and the unmodified control line to investigate the effect of modified phenolic profile on performance of late-instar *E. autumnata* larvae. To take into account the possible induced responses and whole-plant processes, we reared larvae on intact plants, and measured both consumption and growth. Because altering metabolic pathways can introduce unintended effects on plant chemistry and phenotype (Hjältén et al. 2013), we also quantified water and nitrogen content, density of glandular trichomes, specific leaf area and weight, and leaf and plant size. Specifically, we hypothesized that (1) eliminating enzymes along the phenylpropanoid pathway will lead to modification-specific accumulation of upstream phenolics and intermediate compounds, while the concentrations of compounds downstream of the inhibited enzyme will decrease; (2) modification-related changes in leaves will affect both feeding preference and performance of *E. autumnata* larvae; and (3) changes in larval performance can be linked to phenolic compounds conferring resistance to *B. pendula*, or to other leaf parameters affecting diet suitability.

## Methods and Materials

### Plant Material

**Fig. 3:**
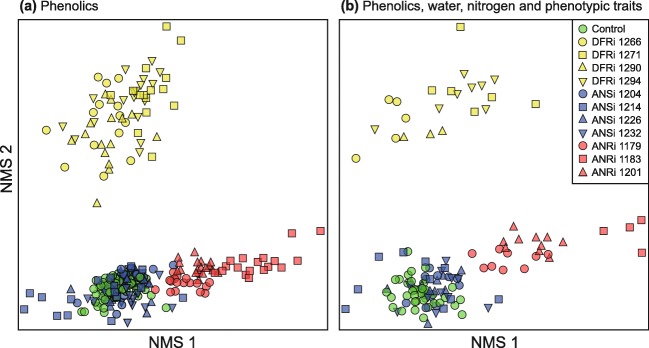
NMS ordination of **(a)** individual experimental plants of the control and modified *Betula pendula* lines (*n* = 260, stress = 9.55) based on concentrations of foliar phenolics and condensed tannins, and **(b)** plants with *Epirrita autumnata* (*n* = 127, stress = 8.27) based on plant chemical and phenotypic traits

We constructed transgenic *B. pendula* RNAi lines with reduced expression of either dihydroflavonol reductase (*DFR*), anthocyanidin synthase (*ANS*) or anthocyanidin reductase (*ANR*) (Fig. 2a). As a recipient, we used the early-flowering clone BPM5 (hereafter the ‘control line’), which is easily transformed and fast-growing (Lemmetyinen et al. 1998). RNAi constructs were generated according to Online Resource Methods S1, and *Agrobacterium* transformation was carried out as described in Kosonen et al. (2015). For each construct, expression of the inhibited enzyme gene was measured with RT-qPCR from 6 to 8 independent transgenic lines (Kosonen et al. 2015), using primers located outside the RNAi construct sequence (Online Resource Methods S2). Foliar mRNA levels decreased to 9.6–31.7% of normal in DFRi, to 1.2–18.9% of normal in ANSi, and to 5.7–47.8% of normal in ANRi lines compared to the expression in control plants (data not shown). Three to four independently transformed lines per RNAi construct (Fig. 3) were included in our experiment.

The control and transformed lines were maintained on Murashige and Skoog (MS) culture (Murashige and Skoog 1962) with 1 mg l^−1^ of 6-benzylaminopurine, and rooted on ½ MS culture with 0.5 mg l^−1^ of IAA. Five weeks after rooting, the plantlets were planted on a 1: 1 mixture of fertilized peat (Kekkilän puutarhaturve, Kekkilä Group) and vermiculite (Vermipu Oy) and transferred to a glasshouse with constant 400 μmol m^−2^ s^−1^ light between 05:00–23:00. Plantlets were planted into 1.55 l of 2: 1 mixture of fertilized peat and vermiculite 13 days before the experiment. After planting, plantlets were fertilized five times with a solution containing 248.6 mg l^−1^ N, 97.4 mg l^−1^ P and 561.41 mg l^−1^ K (Turve-Superex, Kekkilä), with pots retaining on average 220 ml of solution. The rooting of plantlets used in the three experimental replications was scheduled so that, at the onset of each experiment (May 23, May 30, and June 6, 2016), each plant had grown in soil for nine weeks. The plants were put in growth chambers 3–7 h before each experiment.

### Larval Material

The herbivory experiment was conducted with late-instar *E. autumnata* larvae from the second laboratory-grown generation (Online Resource Methods S3) originating from mated females collected from Luftjokdalen in northern Norway (70°14′53” N: 28°23′27″ E) in late August 2014. Newly emerged larvae were collected three times in intervals of ca. seven days (3 × 300 larvae), and larvae of different age classes were reared separately on fresh *B. pubescens* in 10-l plastic buckets with moist garden peat at the bottom until May 13, 2016, after which they were kept at 13 °C in constant darkness, and fed with a mixture of mature *B. pendula* and *B. pubescens* leaves. Before each experimental replication, randomly selected experimental larvae were acclimatized by feeding them fresh leaves of the control line for 16 h at room temperature and natural light, followed by 3 h of starvation before measuring the initial fresh weight (FW_start_) of each larva.

### Experimental Setup

A 48-h feeding experiment was conducted in six growth chambers (Microclima MC1000HE-EVD, Snijder Labs) and repeated with new plants and larvae in three experimental replications performed on consecutive weeks. In total, each experiment included 18–24 plants (of which 12–16 with a larva) per enzyme construct. Each chamber received one plant from each of the 11 RNAi lines and four plants from the control line, with the locations of individual plants randomized within each chamber. Experimental plants were surrounded by additional ‘side plants’ to ensure uniform within-canopy conditions (320 μmol m^−2^ s^−1^ PAR, 23 °C, 55% humidity). The photoperiod was set to 16 h (light): 1 h: 6 h (dark): 1 h, where light intensity was gradually increased or decreased during the intervening 1-h periods. The plants were watered with clean tap water daily.

To investigate whether the presence of herbivores induced changes in foliar phenolics of damaged plants, larvae were not placed on all plants. However, to avoid the possibility of defensive signals spreading from wounded plants to adjacent uninjured ones (Arimura et al. 2000; Moreira et al. 2018), plants with herbivores were assigned to separate chambers from those without herbivores. On each experimental replication, two randomly selected chambers were left without herbivores, while 60 acclimatized and weighed late-instar larvae not starting to molt were randomly placed on plants in the four herbivory chambers, so that each plant received a single larva. Movement of larvae between plants was prevented by mesh bags, closed so that the apical bud and 2–3 youngest leaves were left outside; plants without herbivores were bagged similarly. After placing the larvae on the plants, bags were closed and the larvae were allowed to feed for 48 h before they were removed and put at 13 °C to await further measurements. Chambers were thoroughly cleaned between experimental replications to prevent carry-over effects from larval presence in the earlier replication.

### Measurements of Larvae and Plants, and Chemical Analyses

Immediately after each 48-h experiment, 2–6 undamaged mature leaves from the net-enclosed part of the plant were sampled for chemical and morphological analyses. After recording the FW, the sampled leaves were dry-air dried (relative humidity <20%) according to Tegelberg et al. (2018) and stored at −20 °C. Out of the remaining leaves in each plant, partly eaten leaves were separated and the number of empty petioles (n_pet_) was recorded. Leaf areas eaten (LA_1_) were directly measured when it was possible to estimate the original shape of the leaf; otherwise, areas eaten were estimated by subtracting the remaining leaf areas (LA_r_) from the average area (LA) of the leaves sampled for chemical analyses from the same plant (measured with a LICOR LI-3000C portable area meter; LICOR, Lincoln, NE, USA) as LA_2_ = LA–LA_r_. LA was also used to estimate the area of completely eaten leaves per tree (LA_3_ = n_pet_·LA). Total area eaten per plant (LA_eaten_) was estimated as the sum of these components (LA_eaten_ = LA_1_ + LA_2_ + LA_3_). Specific leaf area (SLA, cm^2^ g^−1^ DW) was estimated by dividing LA of leaves sampled for chemical analyses by their dry weight (DW), and specific leaf weight (SLW, g cm^−2^) as its inverse. The density of glandular trichomes was estimated using a Zeiss Stemi DV4 microscope, by counting leaf glands from a 10% transect extending across the middle of the leaf on both adaxial (upper) and abaxial (lower) surfaces. Remaining leaf biomass and stems were dried at room temperature for measuring total DW.

Post-experiment fresh weight of each larva (FW_end_) was measured the morning after each experimental replication. Larval FWs were converted to DWs using the formula DW = 0.2377*FW–1.5819, which we estimated by weighting 55 larvae with FW between 19 and 100 mg (R^2^ = 0.95). Relative daily consumption rate (RCR) was calculated as RCR = DW_eaten_/(DW_start_ × 2), where DW_eaten_ is the LA_eaten_ converted to DW based on SLA, and relative daily growth rate (RGR) as RGR = (DW_end_–DW_start_)/(DW_start_ × 2). Gross growth efficiency (GGE), measuring the efficiency by which ingested plant biomass is converted to larval biomass, was calculated as GGE = (DW_end_–DW_start_)/DW_eaten_.

To quantify low-molecular weight phenolics in leaves, 5–13 mg samples (or 0.3–5 mg of small leaves) were extracted from ground dry leaves (main leaf vein excluded) following the protocol of Kosonen et al. (2012). Samples were re-dissolved in 0.6 ml of a 1: 1 mixture of MeOH and MilliQ-H_2_O (for smaller samples 0.3 ml was used), and the phenolics were analyzed with HPLC-UV-DAD according to the protocol of Randriamanana et al. (2014), with 10–70 μl injection volumes. D(−)-salicin was used as an internal standard for half of the replicates extracted on each day (mean of daily yields 77.2 ± 1.5%, range 67.4–96.5%; Online Resource Table S1). Compounds were quantified at 220 nm, 280 nm or 320 nm using commercial or purified standards (Online Resource Table S2). Quantified low-molecular weight phenolics were identified with UHPLC-Q-TOF/MS analysis according to Randriamanana et al. (2014). Mass accuracy was calculated as ppm = 10^6^ x (monoisotopic mass − observed mass)/observed mass (Online Resource Table S2).

Acid–butanol assays for condensed tannins (Hagerman 2011) as well as nitrogen analyses (Kosonen et al. 2012) were performed for samples pooled from chemistry and biomass leaves of individual plants. Condensed tannins, purified from leaves of the unmodified control line with the method of Hagerman (2011), were used as a standard for the acid–butanol assays.

### Statistical Analyses

To characterize the phenolics and morphological traits of the plant material and to test if reduced levels of specific enzymes affected the larval parameters, we used linear mixed models in R ver. 3.2.4 (R Core Team 2018) packages lme4 (Bates et al. 2015), lmerTest (Kuznetsova et al. 2017) and nlme (Pinheiro et al. 2018). These models test if RNAi construct, herbivory, and/or their interaction, as specified in the fixed part ***β***^′^***x***_*ijkl*_ (Tables 1 and 2, Online Resource Table S4), affect the response variable *y*_*ijkl*_ in line *i*, experimental replication *j*, chamber *k*, and plant *l*, as

$$ {y}_{ijkl}={\boldsymbol{\beta}}^{\prime }{\boldsymbol{x}}_{ijkl}+{a}_i+{b}_j^{(1)}e{1}_{ijl}+{b}_j^{(2)}e{2}_{ijl}+{b}_j^{(3)}e{3}_{ijl}+{c}_k+{\varepsilon}_{ijkl}. $$

**Table 1:** Summary statistics for the multi- and univariate linear mixed models made for the concentrations of foliar phenolics and condensed tannins in control and modified *Betula pendula* lines, with the fixed effects of RNAi construct, herbivory treatment, and experimental replication

Compound group/Compound^1^	RNAi construct				Herbivory treatment				Experimental replication			
	numDF	denDF	*F*	*P*	numDF	denDF	*F*	*P*	numDF	denDF	*F*	*P*
Phenolic acids	9	772	391.59	<0.001	3	772	0.23	0.874	6	772	14.19	<0.001
Log_e_(*p*-OH-cinnamic acid monoglucoside +10)	3	8	17.38	<0.001					2	253	4.94	0.008
Log_e_ (coumaroylquinic acid derivative 1 + 0.1)	3	8	1130.71	<0.001					2	253	12.79	<0.001
coumaroylquinic acid derivative 2	3	8	9.28	0.006					2	253	5.58	0.004
Flavones	6	511	10.51	<0.001	2	511	1.46	0.234	4	511	25.15	<0.001
flavone 1	3	8	0.99	0.444					2	253	28.31	<0.001
sqrt (flavone 2)	3	8	20.48	<0.001					2	253	32.80	<0.001
Dihydroflavonols	9	772	57.39	<0.001	3	772	0.40	0.757	6	772	12.47	<0.001
Log_e_ (ampelopsin monoglucoside+0.1)	3	8	149.71	<0.001					2	253	24.05	<0.001
sqrt (taxifolin monoglucoside)	3	8	106.19	<0.001					2	253	23.83	<0.001
sqrt (dihydroflavonol 3)	3	8	42.63	<0.001					2	253	10.78	<0.001
Myricetins (flavonols)	15	1294	91.83	<0.001	5	1294	0.54	0.748	10	1294	7.18	<0.001
Log_e_ (myricetin 3-galactoside)	3	8	30.66	<0.001					2	253	6.48	0.002
sqrt (myricetin 3-glucoside)	3	8	67.75	<0.001					2	253	4.13	0.017
Log_e_ (myricetin 3-arabinoside +10)	3	8	15.53	0.001					2	253	3.16	0.044
myricetin 3-rhamnoside	3	8	6.68	0.014					2	253	8.28	<0.001
myricetin methyl ether 3-glucoside	3	8	14.49	0.001					2	253	8.89	<0.001
Quercetins (flavonols)	9	772	24.02	<0.001	3	772	0.54	0.652	6	772	5.80	<0.001
quercetin 3-galactoside	3	8	5.14	0.029					2	253	6.16	0.002
quercetin 3-glucoside	3	8	9.78	0.005					2	253	0.32	0.724
quercetin 3-rhamnoside	3	8	2.20	0.165					2	253	3.84	0.022
Other compounds												
sqrt (kaempferol 3-rhamnoside)	3	8	2.91	0.101	1	253	3.49	0.063	2	253	4.47	0.012
Log_e_ (catechin +0.1)	3	8	240.83	<0.001	1	253	1.69	0.194	2	253	72.74	<0.001
unidentified 1	3	8	0.38	0.773	1	253	0.03	0.866	2	253	3.58	0.029
DPPG	3	8	0.39	0.767	1	253	0.92	0.338	2	253	5.28	0.006
Log_e_ (condensed tannins)	3	8	4.59	0.038	1	245	3.70	0.056	2	245	17.55	<0.001

numDF, numerator degrees of freedom; denDF, denominator degrees of freedom;

^1^The fixed part of the model included RNAi construct (control, DFRi, ANSi, or ANRi), herbivory treatment, and experimental replication, and the model included random intercepts for plant lines. Lines in bold refer to overall tests for each whole group of compounds, while other lines show results for individual compounds within each group. Other compounds were tested with corresponding univariate models, and compounds not present in the control line were omitted from the models. The models for low-molecular weight phenolics were based on observations from 268 plants, and for condensed tannins on observations from 260 plants

**Table 2:** Summary statistics from linear mixed models made for *Epirrita autumnata* consumption and growth parameters, with RNAi construct as a fixed factor and larval starting weight as a covariate

Larval trait	RNAi construct				larval starting weight			
	numDF	denDF	*F*	*P*	numDF	denDF	*F*	*P*
Log_e_ (consumption+10)^1^	1	5.6	11.21	0.009	1	13.7	8.54	0.011
Log_e_ (RCR + 0.1)^1^	3	2.0	7.12	0.126	1	13.7	2.27	0.132
growth^2^	3	2.0	4.42	0.190	1	96.3	23.01	<0.001
Log_e_ (RGR + 1)^1^	3	2.2	3.73	0.202	1	23.5	303.52	<0.001
Log_e_ (GGE+100)^1^	3	4.7	2.03	0.236	1	10.6	15.88	0.002

numDF, numerator degrees of freedom; denDF, denominator degrees of freedom; RCR, relative daily consumption rate; RGR, relative daily growth rate; GGE, gross growth efficiency

^1^The models based on 138 (GGE) or on 149 (other variables) observations of plants and larvae included random intercepts for plant lines, for each combination of experimental replication and RNAi construct, and for chambers

^2^The model based on 149 observations of plant and larva included random intercepts for plant line, experimental replication (Online Resource Fig. S2), and chamber

Dependency of observations belonging to the same line (*a*_*i*_) or chamber (*c*_*k*_) was considered in the models by including corresponding random intercepts. The effect related to experimental replication was enabled to vary within each construct by including construct-specific random intercepts (${b}_j^{(1)},{b}_j^{(2)},{b}_j^{(3)}$; modified by binary terms *e*1_*ijl*_, *e*2_*ijl*_, and *e*3_*ijl*_ which connect each observation to its respective construct). Random effects and model residuals *ε*_*ijkl*_ were assumed to be normally distributed with a mean of zero and to have constant variance among groups. Suitable transformations (Tables 1 and 2, Online Resource Table S4) for response variables were selected based on graphs of model residuals. For significant fixed effects, Holm-adjusted contrasts were used as post-hoc tests to test which levels differed from the control. When modeling phenolics, multivariate mixed-effect models (Online Resource Methods S4) were used to allow hypothesis testing on the whole groups of compounds produced by the same step of the flavonoid pathway (Fig. 2a), as well as on individual compounds. As the nlme package does not allow a crossed design of random effects, experimental replication was included as a fixed effect. Interactive effects of replication and RNAi construct on phenolics (summarized in Online Resource Table S3) were excluded from final models (Table 1). When modeling leaf nitrogen and water content and plant phenotypic traits (Online Resource Table S4), the effect of herbivory was excluded when it did not significantly affect model fit (*P* > 0.1 from conditional Wald’s *F*-tests). Larval starting weight was included as covariate when modeling consumption and growth parameters of larvae (Table 2).

Variation in chemical profiles of individual experimental plants was visualized using non-parametric multidimensional scaling (NMS) ordination in PC-ORD ver. 7.04 (McCune and Mefford 2016). Compound-specific concentrations were transformed to relative scale by dividing them by their maxima. NMS based on Sorensen distances was run first with the slow-and-thorough autopilot method of PC-ORD (no penalty for tie removal), after which varimax rotation was applied. Blocked multi-response permutation procedures (MRBP) in PC-ORD, based on replication-specific averages within RNAi constructs and using median alignment between blocks, were used to test for differences in overall chemistry among constructs. Differences in overall chemistry among lines within RNAi constructs were studied separately using multi-response permutation procedures (MRPP) in PC-ORD, with Euclidean distances and weighting according to Mielke (1984). Corresponding MRBP and MRPP tests were also done using a dataset including only foliar nitrogen and water content and plant phenotypic traits specified in in Table 3.

**Table 3:** Correlation coefficients and Bonferroni-adjusted significances of foliar phenolics, condensed tannins, and water and nitrogen content, and *Betula pendula* phenotypic traits with NMS axis scores in Fig. 3b

Leaf phenolics	Kendall’s tau with NMS1		Kendall’s tau with NMS2	
*Phenolic acids*				
p-OH cinnamic acid monoglucoside	0.11		−0.42	***
coumaroylquinic acid derivative 1	0.28	***	−0.14	
coumaroylquinic acid derivative 2	−0.01		−0.48	***
*Flavanones*				
flavanone 1	0.02		0.53	***
flavanone 2	0.01		0.53	***
flavanone 3	0.12		0.46	***
*Flavones*				
flavone 1	0.38	***	−0.02	
flavone 2	0.34	***	0.34	***
*Dihydroflavonols*				
dihydroflavonol 1	0.02		0.55	***
ampelopsin diglucoside	0.05		0.48	***
ampelopsin monoglucoside	0.21		0.55	***
ampelopsin	0.02		0.53	***
dihydroflavonol 2	0.00		0.50	***
taxifolin monoglucoside	0.04		0.37	***
taxifolin	0.03		0.54	***
dihydroflavonol 3	0.04		0.26	**
*Flavonols*				
*Myricetins*				
myricetin 3-galactoside	0.61	***	0.16	
myricetin 3-glucoside	0.61	***	0.30	***
myricetin 3-arabinoside	0.53	***	−0.07	
myricetin 3-rhamnoside	0.40	***	0.36	***
myricetin methyl ether 3-glucoside	0.38	***	0.46	***
*Quercetins*				
quercetin 3-galactoside	0.34	***	−0.29	***
quercetin 3-glucoside	0.55	***	0.03	
quercetin 3-arabinoside	0.57	***	0.47	***
quercetin 3-rhamnoside	0.34	***	0.24	**
*Other flavonols*				
kaempferol 3-rhamnoside + phenolic acid	0.02		−0.38	***
*Flavan-3-ols*				
gallocatechin	−0.11		−0.26	*
catechin	−0.06		−0.38	***
*Other phenolics*				
unidentified 1	−0.03		−0.18	
DPPG	0.15		−0.02	
*Condensed tannins*	−0.05		−0.34	***
Nitrogen, water and phenotypic traits	Kendall’s tau with NMS1		Kendall’s tau with NMS2	
leaf area	−0.37	***	−0.74	***
leaf dry weight	−0.26	**	−0.79	***
leaf fresh weight	−0.32	***	−0.76	***
adaxial gland density	0.36	***	0.28	***
abaxial gland density	0.40	***	0.20	
leaf water content	−0.37	***	−0.24	**
leaf nitrogen content	−0.20		−0.27	***
specific leaf area	−0.48	***	−0.25	**
specific leaf weight	0.48	***	0.25	**
plant leaf biomass	−0.18		−0.77	***
plant stem biomass	−0.19		−0.78	***
plant aboveground biomass	−0.18		−0.78	***

* *P* < 0.05; ** *P* < 0.01; ***, *P* < 0.001

To discern which chemical or phenotypic traits in plants influence larval consumption and performance parameters, we first constructed a separate NMS ordination (using the aforementioned distance and relativization settings) based on combined chemical and phenotypic data (variables in Table 3). This ordination resulted in a 2-dimensional solution (Fig. 3b) largely corresponding to the results from the NMS ordination based solely on foliar phenolics (Fig. 3a). We then used sample coordinates along the two NMS axes and larval starting weight as predictors in linear mixed models explaining larval parameters (Online Resource Table S5), with random parts as above, except that construct-specific intercepts for replication were replaced by a general intercept for replication (*b*_*j*_). Kendall’s rank correlation between NMS scores and plant variables (Table 3) were used to characterize the relationship between NMS axis coordinates and plant chemical and phenotypic traits.

## Results

### Effects of RNA Interference on Leaf Phenolics and Other Plant Traits

The quantified low-molecular weight phenolics included 29 compounds, out of which 21 could be identified with UHPLC-QTOF/MS (Online Resource Table S2). Leaves of the unmodified control line contained 18.9 ± 2.4 mg g^−1^ (mean ± SE) of low-molecular weight phenolics (mainly DPPG, and rhamnosides of myricetin and quercetin) and 85.3 ± 17.8 mg g^−1^ of condensed tannins. Reducing the expression of the three focal enzymes of the flavonoid pathway changed the absolute and relative concentrations of different phenolic compounds in a construct-specific manner (Table 1, Fig. 2). Most changes were quantitative, but some phenolics found in the control line disappeared, and a few new phenolics were introduced in the RNAi constructs (Online Resource Table S3). Reflecting these coordinated changes in foliar phenolic chemistry, individual experimental plants were clearly grouped according to RNAi construct in two-dimensional NMS ordination space (Fig. 3a; MRBP, *P* = 0.009). While distances among lines within constructs were smaller (Fig. 3a), lines differed statistically significantly in all three construct-specific MRPP tests (all *P* < 0.01). Differences among experimental replications were found for most individual compounds, but the effect was small in comparison to the effect of RNAi construct (Table 1).

The clearest changes in phenolic chemistry were observed in DFRi plants (Fig. 3a), in which the concentrations of flavanones (Fig. 2c; not present in the control line) and dihydroflavonols (*P* < 0.001; Fig. 2e) increased. The total concentration of the latter increased nearly a thousand-fold compared to the control line, and this consisted mainly of ampelopsin (also known as dihydromyricetin), present at 19.2 ± 5.7 mg g^−1^ in the leaves of DFRi plants (Online Resource Table S3). By contrast, no foliar gallocatechin or catechin were observed in these plants. The concentration of condensed tannins was reduced by 55% (*P* = 0.038; Fig. 2i) and that of total phenolic acids by 78% (*P* < 0.001; Fig. 2b).

Inhibiting *ANS* expression increased the concentration of foliar catechin by 316% (*P* < 0.001; Fig. 2h). Also, small amounts of gallocatechin, not observed in the control line, accumulated in ANSi plants (Online Resource Table S3).

The main effect of ANRi was an increased concentration of certain flavonols (Fig. 2f,g), especially myricetin 3-galactoside and myricetin 3-glucoside (*P* < 0.001 for both compounds). Quercetin 3-arabinoside, not observed among the foliar phenolics of the control line, accumulated in minor concentrations in ANRi lines (Online Resource Table S3).

Phenotypic effects of RNA interference were limited to DFRi and ANRi plants (Online Resource Fig. S1, Table S4), which were smaller (*P* < 0.001 for leaf, stem and aboveground biomass, Online Resource Fig. S1i,j,k) and had smaller leaves than control plants (*P* < 0.001 for LA and DW of DFRi, and *P* = 0.029 for DW of ANRi plants, Online Resource Fig. S1a,b). Statistically marginally significant differences among RNAi constructs were found for leaf water (74.1 ± 2.2%; *P* = 0.080) and nitrogen content (4.1 ± 0.3%; *P* = 0.058), apparently due to slightly lowered values in DFRi and ANRi plants (Online Resource Fig. S1e,f). Inhibiting enzyme expression did not affect SLA (260 ± 20 cm^2^ g^−1^; *P* = 0.654) or specific leaf weight (SLW; 4.0 ± 0.4 mg cm^−2^; *P* = 0.710; Online Resource Fig. S1 g,h). A multivariate analysis done solely for phenotypic traits and foliar water and nitrogen content (results not shown) revealed a statistically significant overall difference among RNAi constructs (*P* = 0.006 in MRBP). Lines within DFRi and ANSi constructs differed (MRPP, *P* = 0.003 and *P* = 0.005, respectively) but no effect of line was found within ANRi (*P* = 0.197).

### Effects of Larval Feeding on Plant Chemistry and Phenotype

The presence of a single *E. autumnata* larva feeding on a plant for 48 h did not induce changes in phenolic chemistry in undamaged leaves of the same plant (*P* > 0.05 for all treatment effects; Table 1). Likewise, exposure to herbivory had no effect on most of the measured non-phenolic plant traits (Online Resource Table S4, Online Resource Fig. S1). A slight interactive effect of herbivory and RNAi modification was found for LA and gland densities on both leaf surfaces, but in Holm-adjusted contrasts, the only statistically significant difference between treatments within an RNAi construct was in adaxial gland density, which was 21% higher in ANRi plants exposed to herbivores than in plants in control chambers (*P* = 0.019; Online Resource Fig. S1c). The increase could be caused by slightly smaller leaves in those plants (Online Resource Fig. S1a), even though the difference in LA between treatments within ANRi plants was statistically only marginally significant (*P* = 0.055).

### Consumption and Performance of Larvae on Control and RNAi Lines

**Fig. 4:**
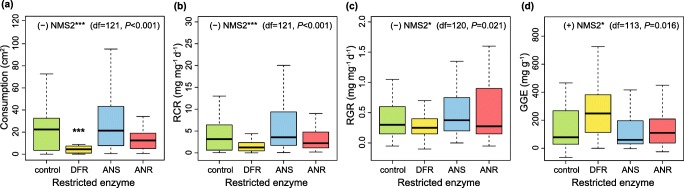
Effect of RNAi constructs on **(a)** consumption, **(b)** relative consumption rate (RCR), **(c)** relative growth rate (RGR) and **(d)** gross growth efficiency (GGE) of *Epirrita autumnata* larvae, with medians and 95% confidence intervals shown. Asterisks over boxes denote Holm-adjusted differences to the control line at 0.01 < *P* < 0.05 (*), 0.001 < *P* < 0.01 (**), or *P* < 0.001 (***). Inset texts refer to significant effects of NMS axis scores (Fig. **3****b**) on the focal parameters according to explanatory models (Online Resource Table S5)

On average, larvae consumed 29.1 ± 17.4 cm^2^ of leaves of the control line, and larval weight increased by 5.7 ± 2.1 mg DW (23.8 ± 9.1 mg FW) during 48 h. RNAi construct had a statistically significant effect on absolute leaf consumption (Table 2) when controlling for larval starting weight. However, while inhibition of DFR decreased absolute consumption by 82% (*P* < 0.001 from Holm-adjusted contrasts), ANSi or ANRi had no effect on larval feeding (Fig. 4a). In contrast to absolute consumption, larval RCR (on average, 5.8 ± 6.8 mg mg^−1^ d^−1^; Fig. 4b), absolute growth, RGR (on average, 0.46 ± 0.28 mg mg^−1^ d^−1^, Fig. 4c), and GGE (on average, 280 ± 250 mg g^−1^, Fig. 4d) did not differ among RNAi constructs (Table 2).

Linear mixed models in which larval consumption and growth parameters were explained by the overall chemical and phenotypic properties of the experimental plants (Fig. 3b) revealed that absolute and relative leaf consumption, and relative growth rate were highest on plants with low scores along Axis 2 of the NMS ordination (*P* < 0.001 for both consumption parameters, *P* = 0.021 for RGR; Fig. 4a–c; Online Resource Table S5). While larval absolute growth was not influenced by the NMS axis scores, a positive relationship between GGE and NMS Axis 2 scores (NMS2) was found (*P* = 0.016; Online Resource Table S5).

Compounds potentially associated with lower absolute and relative feeding and relative growth include flavanones, dihydroflavonols, and some flavones, myricetins and quercetins; additionally, larvae generally fed and grew (relative to their starting weight) less on leaves with high adaxial glandular trichome density, or a high SLW (positive correlation with NMS2 in Fig. 3b, Table 3). NMS2 also correlated positively with the overall concentration of low-molecular weight phenolics in leaves (Kendall’s tau 0.45, Bonferroni-adjusted *P* < 0.001), implying a negative connection with larval absolute feeding, RCR and RGR. Compounds potentially associated with low larval gross growth efficiency include some phenolic acids, kaempferols, quercetin 3-galactoside, flavan-3-ols, and condensed tannins, and larval GGE was lowest on plants with large leaves with high SLA, nitrogen or water content (negative correlation with NMS2 in Fig. 3b; Table 3).

## Discussion

Molecular-genetic methods for up- or downregulation of gene expression in plants provide numerous opportunities for experimental manipulation of plant biochemistry (Hammerbacher et al. 2019; Tian et al. 2015), but also for investigating the effects of specific plant compounds on feeding behavior and performance of associated herbivores (Kosonen et al. 2012; Müller et al. 2015; Sasse et al. 2016). In our study, inhibition of DFR and ANR activity altered the chemo- and phenotype of young *B. pendula*. The changes occurring in DFRi plants decreased absolute and relative consumption and relative growth, but increased gross growth efficiency of *E. autumnata* larvae. Below, we evaluate how larval behavioral responses to diet quality and various plant-related variables explain the observed differences in larval consumption and growth, and relate our results to previous work on plant defensive chemistry.

RNAi silencing of the three target genes produced chemical changes that comply with the predicted decreases in phenolics downstream of, and accumulation of compounds upstream of, each inhibited enzyme (Fig. 2). As expected, the clearest chemical changes occurred in DFRi plants, in which the levels of low-molecular weight flavonoids produced prior to DFR were elevated, evidently at the expense of flavan-3-ols and condensed tannins (Fig. 2). Corresponding phytochemical changes have been observed in DFR-deficient *Arabidopsis thaliana* (Routaboul et al. 2006), *Nicotiana tabacum* (Lim et al. 2016) and *Malus* spp. (Tian et al. 2015). Conversely, levels of phenolic glycosides are lowered in transgenic *Populus* lines in which production of condensed tannins has been upregulated by overexpression of the MYB134 transcription factor (Kosonen et al. 2012). Taken together, these results support the existence of trade-offs in the allocation of resources to alternative end products along the flavonoid pathway (Wam et al. 2017; Winkel-Shirley 2002b).

Silencing of *ANS* and *ANR* led to less drastic shifts in foliar chemistry, mainly seen as increases in the levels of several flavonols in ANRi and in catechin in ANSi lines (Fig. 2). In accordance with earlier work, ANRi birches also exhibited a dark reddish color (Fischer et al. 2014; Kosonen et al. 2015; Kovinich et al. 2012;). While the concentration of condensed tannins was not altered (Fig. 2i), the mean degree of polymerization was lowered in the mixture of condensed tannins typical to ANSi or ANRi lines, and their average catechin: epicatechin ratios increased compared to the condensed tannins in the control line (Thitz et al. unpublished results). This indicates channeling of substrates towards catechin from flavan-3,4-diols (Fig. 2a,h) in *B. pendula* with downregulated *ANS* and *ANR*.

Disruption of the flavonoid pathway at ANS produced hardly any phenotypic changes, but plant growth was severely retarded in ANRi and DFRi plants (Online Resource Fig. S1). The proposed structural role of condensed tannins in plant cell walls (Cholet et al. 2014; Keski-Saari et al. 2007; Pizzi and Cameron 1986) could explain the stunted growth of the low-tannin DFRi plants. Alternatively, reduced growth in the DFRi and ANRi lines could be caused by detrimental interactions of flavonoids with growth-promoting phytohormones (Besseau et al. 2007; Brown et al. 2001), or by direct harmful effects of flavonols (Harding 2019; Sakihama 2002). While the concentration of foliar nitrogen was slightly lowered in DFRi and ANRi plants (Online Resource Fig. S1f), it still remained above the 1–3.5% typical of field-grown *B. pendula* (Martemyanov et al. 2015; Percival and Smiley 2015; Sellin et al. 2013), which makes nitrogen limitation an unlikely candidate for the decreased growth of these lines. Evidently, elucidating the role of phytochemicals in relation to plant growth deserves further study.

Research on plant defenses is complicated by inconsistent terminology and inferences across studies: for example, plant ‘resistance’ can be measured as herbivore growth (Mutikainen et al. 2002)—which may or may not correlate with the amount of feeding damage inflicted—, and herbivore ‘performance’ is sometimes used synonymously with consumption (Leimu et al. 2005)—which is not necessarily correlated with herbivore growth or other fitness parameters. Additionally, a defensive compound is beneficial for the plant only if it reduces feeding or replacement cost of lost tissues (Augner 1995), which may not be the case if herbivores compensate for poor dietary quality by increasing consumption (Fig. 1; Karban and Agrawal 2002; Lee 2017; Simpson and Simpson 1990).

The combination of trade-offs in the production of different compounds in plants and plastic feeding responses in herbivores means that multiple interconnected factors need to be considered simultaneously when interpreting results from feeding experiments (Fig. 1). In our study, *E. autumnata* larvae consumed less of DFRi plants as compared to the control, ANRi and ANSi plants, which led to slightly lower larval RGR on the same plants. Such a pattern points to the presence of feeding deterrents in DFRi plants, as compared to the control, ANSi and ANRi lines (Fig. 1). However, the reduction in larval consumption was proportionally larger than the decrease in larval growth, resulting in a statistically significant positive connection between GGE and NMS2 (Fig. 3b, inset in Fig. 4d). This points to the conclusion that DFRi plants were, in terms of physiological suitability, of the best quality for the experimental larvae (Fig. 1).

Which characteristics of DFRi plants explain the reduced consumption and growth, but improved growth efficiency of *E. autumnata* larvae on them? In NMS ordination space (Fig. 3), DFRi is separated from the control and ANSi/ANRi constructs mainly along Axis 2, which largely corresponds to a chemical shift from the production of flavan-3-ols and condensed tannins towards flavanones, dihydroflavonols and several flavonol glycosides, but also to an increase in the density of adaxial glandular trichomes and a decrease in plant size, nitrogen and water content (Table 3). Since there seems to be little reason to suspect direct effects of plant or leaf size on herbivore feeding or growth (smaller leaves may force the larvae to move more, but on smaller plants the distances between leaves are also shorter), and the highest larval GGE (indicating best diet quality from a purely physiological perspective) was observed on plants with the lowest nitrogen and water contents, plant size and nutrients appear to be unlikely drivers of the larval responses in our experiment.

It is tempting to suggest antiherbivory functions for structurally highly variable groups of compounds such as low-molecular weight flavonoids (e.g. Harborne and Williams 2000; War et al. 2012), which are abundant across the plant kingdom (e.g. Cheynier et al. 2013). However, studies on the bioactivity of flavonol glycosides against insect herbivores feeding on silver birch (Mutikainen et al. 2000; Peltonen et al. 2010) and other plants (e.g. Nykänen and Koricheva 2004; Simmonds 2003; Treutter 2005) have produced conflicting results. In our experiment, *E. autumnata* larvae consumed less of the flavonoid-rich DFRi lines, and the concomitant decrease in RGR suggests that these compounds act as feeding deterrents (Figs. 1, 4). Alternatively, the observed feeding deterrence could be caused by the relatively high density of glandular trichomes in DFRi lines, as foliar glands have previously been linked to decreased *E. autumnata* RGR on *B. pubescens* ssp. *pumila* (Valkama et al. 2005b). However, glandular trichomes are closely associated with flavonoid aglycones on leaf surfaces of *B. pendula*, (Valkama et al. 2005b), so it is possible that levels of intracellular flavonoids also correlate with the density of leaf glands and their biological activity. Regardless of the foliar location of feeding-deterrent flavonoids, their bioactivity is likely to be mediated by their interactions with polyphenol oxidases (PPOs), known to be activated during defensive responses of *Betula* species against *E. autumnata* (Ruuhola et al. 2008).

While in our experiment larval feeding and RGR were higher on plants with low scores along NMS2, growth efficiency of larvae changed in the opposite direction (Fig. 4). Low GGE therefore appears to be associated with high concentrations of most phenolic acids, quercetin 3-galactoside, flavan-3-ols and condensed tannins, all of which are negatively correlated with NMS2 (Table 3). Since no growth-reducing effects on *E. autumnata* have been attributed to coumaroylquinic acids (composing the bulk of phenolic acids in our experimental plants; Fig. 2b) or quercetin 3-galactoside in *Betula* species (Haviola et al. 2007; Peltonen et al. 2010; Ruuhola et al. 2007), condensed tannins or their flavan-3-ol subunits seem the most likely variables reducing larval GGE in our experiment.

Condensed tannins have been shown to reduce herbivore growth, growth efficiency, pupal mass and/or survival in many plant–insect systems (e.g. Ayres et al. 1997; Bryant et al. 1993; Haviola et al. 2007; Mutikainen et al. 2000; Ossipov et al. 2001), and to frequently induce compensatory feeding (Boeckler et al. 2014; Osier et al. 2000). Plant tissues may contain up to 25% of condensed tannins, so it has been suggested that poor herbivore growth on tannin-rich plants could in some cases reflect simple dilution of limiting nutrients (reviewed in Barbehenn and Constabel 2011). Direct nutrient dilution seems an unlikely explanation for our results because the highest levels of both nitrogen and condensed tannins occurred in the same plants. Instead, the comparatively low larval GGE on high-tannin plants (inset in Fig. 4d, Table 3) supports the idea that condensed tannins of *B. pendula* are either antinutritive or toxic for *E. autumnata.* Antinutritive effects of condensed tannins relate to their interactions with proteins, which reduce nitrogen availability for herbivores (Lattanzio et al. 2006; War et al. 2012), whereas toxic effects could be linked to their spontaneous or PPO-mediated oxidation reactions (Salminen and Karonen 2011).

Currently, it is widely recognized that not all insect herbivores are sensitive to condensed tannins (Barbehenn and Constabel 2011). Structural differences among condensed tannins, as well as specific adaptations in some herbivores, may explain variable tannin activity in different systems (Ayres et al. 1997; Forkner et al. 2004). In our study, *E. autumnata* did not respond to changes in the composition of condensed tannins: neither growth nor GGE differed between the control and ANSi/ANRi lines (Fig. 4c,d) even though mean polymer size and proportion of epicatechin-type subunits were clearly decreased in the mixture of condensed tannin typical of the latter constructs, as mentioned above. To differentiate between antinutritive and toxic activities of condensed tannins against folivorous larvae, the structure–activity relationships of various condensed tannins should be studied in tannin-sensitive insect species. As our results show, genetic manipulation of flavonoid pathway in plants can effectively be used to produce altered mixtures of condensed tannins required for such studies.

Research on the chemical ecology of plant–herbivore interactions is complicated by the combination of trade-offs in the production of different compounds in plants and phenotypic plasticity in the insects that feed on them. Decreased consumption of *E. autumnata* larvae on high-flavonoid, low-tannin *B. pendula* RNAi plants implies that small flavonoids act as feeding deterrents against the larvae. However, the phenolic compounds most strongly linked with decreased diet quality (measured as decreased GGE) were condensed tannins and their monomeric flavan-3-ol subunits. The dose–response relationships of various low-molecular weight phenolics and condensed tannins in generalist and specialist herbivores, as well as the role of these compounds for normal plant development, clearly deserve further study.

## Electronic supplementary material

ESM 1 (PDF 4547 kb)

## Data Availability

A schematic illustration of the experimental setup, primary data, R scripts and parameter estimates of models will be deposited in the Dryad repository (datadryad.org) upon manuscript acceptance.
