# Supplementary material for: Phytochemical Shift from Condensed Tannins to Flavonoids in Transgenic Betula pendula Decreases Consumption and Growth but Improves Growth Efficiency of Epirrita autumnata Larvae
Source: J Chem Ecol. 2019 Dec 26;46(2):217–31. doi: 10.1007/s10886-019-01134-9 (PMC7056695; doi:10.1007/s10886-019-01134-9)
Supplement: Supplementary file 1 — (PDF 4547 kb) [file 10886_2019_1134_MOESM1_ESM.pdf]

Electronic Supplementary material

Article title: Phytochemical Shift from Condensed Tannins to Flavonoids in Transgenic *Betula pendula* Decreases Consumption and Growth but Improves Growth Efficiency of *Epirrita autumnata* Larvae
Journal name: Journal of Chemical Ecology
Authors: Thitz, P., Mehtätalo, L., Välimäki, P., Randriamanana, T., Lännenpää, M., Hagerman, A.E., Andersson, T., Julkunen-Tiitto, R., Nyman, T.
Corresponding author: Thitz, Paula. Department of Environmental and Biological Sciences, University of Eastern Finland; email: paula.thitz@uef.fi

The following Electronic Supplementary Material is available for this article:

Fig. S1 Effects of RNAi and *Epirrita autumnata* treatment on *Betula pendula* parameters
Fig. S2 Effect of experimental replication on consumption and growth of *Epirrita autumnata*
Fig. S3 Comparison of *Betula pendula* DFR and ANS amino acid sequences with corresponding sequences in other species
Table S1 Daily yields of internal standard recovered
Table S2 Identification and quantification of low-molecular weight phenolics
Table S3 *Betula pendula* foliar phenolics in different RNAi constructs and replications
Table S4 Summary statistics for plant trait models
Table S5 Summary statistics for explanatory larval models
Methods S1 Generating *Betula pendula* constructs with decreased expression of *DFR*, *ANS* or *ANR*
Methods S2 Primers used in the qRT-PCR tests of *DFR*, *ANS* and *ANR*
Methods S3 Rearing conditions of *Epirrita autumnata* in the laboratory
Methods S4 Multivariate linear mixed effect models for testing the effects on leaf phenolics

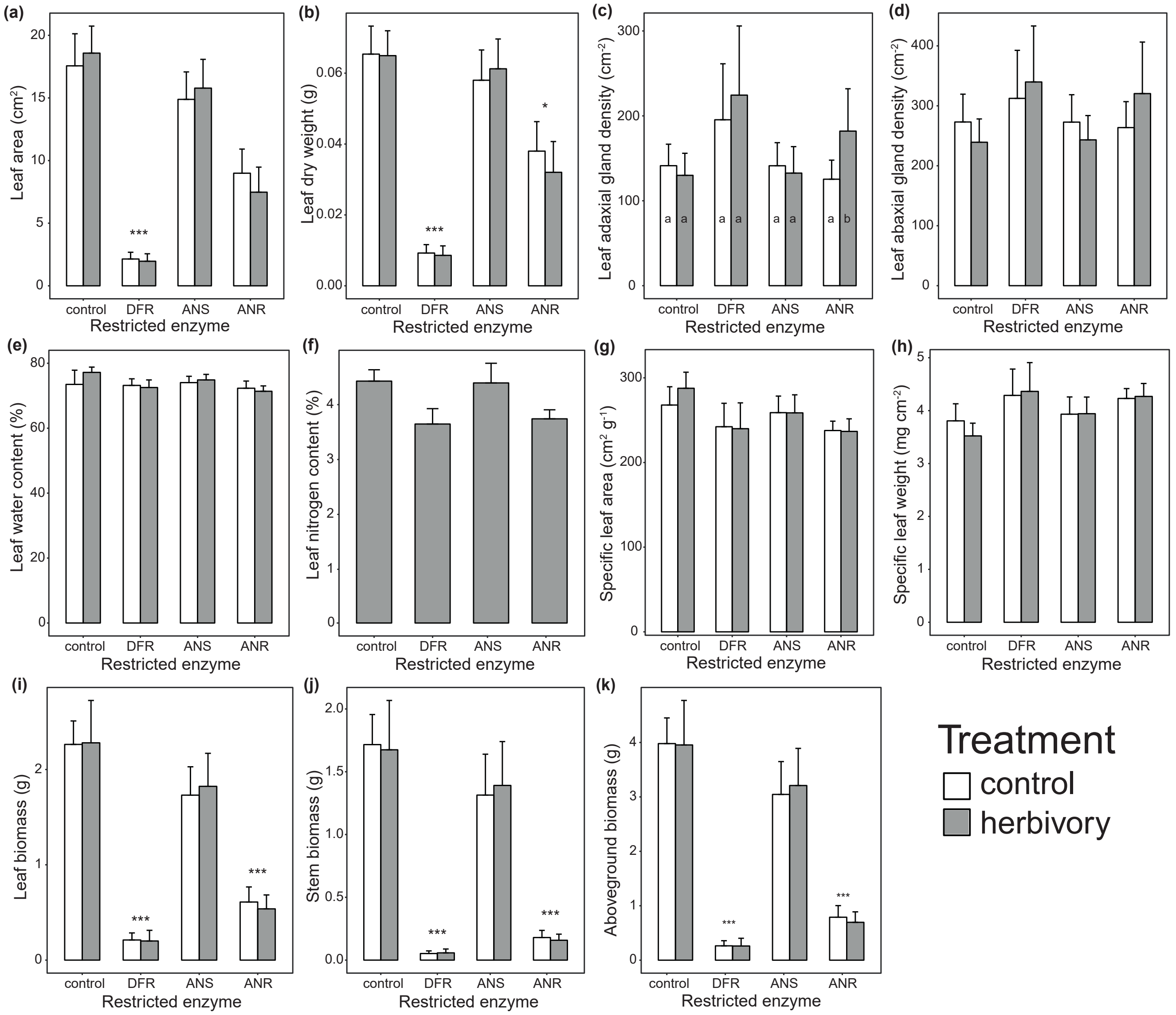

Fig. S1 Effects of RNAi constructs and larval treatments on phenotypic traits and nitrogen and water content in the leaves of control and modified *Betula pendula* plants. Means and ± 1 SE are shown. Asterisks denote differences between the RNAi construct and the control line at Holm-adjusted *P*-values (0.01<*P*<0.05 (*), 0.001<*P*<0.01 (**), or *P*<0.001 (***)). Different letters on bars denote significant differences (*P*<0.05) between plants in different treatments within an RNAi construct.

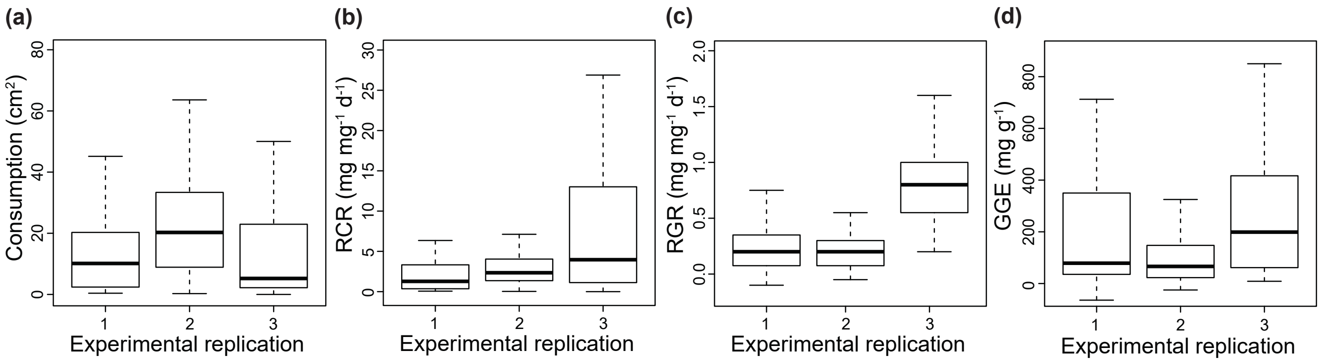

Fig. S2 (a) Consumption, (b) relative consumption rate (RCR), (c) relative growth rate (RGR) and (d) gross growth efficiency (GGE) of *Epirrita autumnata* larvae in the three experimental replications, with medians and 95% confidence intervals shown.

(a)

```
BpANS          1 --MVTSVAPRVESLSSSGIQAIPKEYVRPQEELNSIGNIFDEEKKEEGPQVPTIDLKNIE
VitisANS       1 --MVTSVAPRVESLSSSGIQSIPKEYIRPQEELTSIGNVFEEEKKDEGPQVPTIDLKDIE
ArabANS        1 ----MVAVERVESLAKSGIISIPKEYIRPKEELESINDVFLEEKKEDGPQVPTIDLKNIE
FragariaANS    1 MVTAASIGSRVESLASSGISTIPKEYVRPEEELVNIGDIFEDEKSTEGPQVPTIDLKEID

BpANS         59 SEDQAVREKCREELKRAAVEWGVMHLVNHGISDELLERVKKAGKVFFDLPVEEKEKYAND
VitisANS      59 SEDEVVRERCREELKKAAMEWGVMHLVNHGISDDLINRVKVAGETFFNLPIEEKEKYAND
ArabANS       57 SDDEKIRENCIEELKKASLDWGVMHLINHGIPADLMERVKKAGEEFFSLSVEEKEKYAND
FragariaANS   61 SDDIKVREKCREDLKEAAVEWGVMHLINHGISDELMERVKKAGKAFFDLPIEQKEKYAND

BpANS        119 QALGKIQGYGSKLANNASGQLEWEDYFFHLVYPEDKRDLSIWPKTPTDYIVATTEYAKQL
VitisANS     119 QASGMIAGYGSKLANNASGQLEWEDYFFHLIFPEDKRDMTIWPKTPSDYIPATCEYSVKL
ArabANS      117 QATGKIQGYGSKLANNASGQLEWEDYFFHLAYPEEKRDLSIWPKT-SDYIEATSEYAKCL
FragariaANS  121 QASGKIQGYGSKLANNASGQLEWEDYFFHCVYPEDKRDLSIWPQTPSDYIVATSEYAKEL

BpANS        179 RILASKVFSVLSLGLGLEEGRLEKEVGGLEELLLQMKINYYPICPQPELALGVEAHTDVS
VitisANS     179 RSLATKILSVLSLGLGLEEGRLEKEVGGMEELLLQKKINYYPKCPQPELALGVEAHTDVS
ArabANS      176 RLLATKVFKALSVGLGLEPDRLEKEVGGLEELLLQMKINYYPKCPQPELALGVEAHTDVS
FragariaANS  181 RGLATKILSILSLGLGLEEGRLEKEVGGLEELLLQMKINYYPKCPQPELALGVEAHTDIS

BpANS        239 ALTFILHNMVPGLQLFYEGKWVTAKCVPNSIIMHIGDTLEILSNRKYKSILHRGLVNKEK
VitisANS     239 ALTFILHNMVPGLQLFYEGKWVTAKCVPNSIIMHIGDTIEILSNGKYKSILHRGLVNKEK
ArabANS      236 ALTFILHNMVPGLQLFYEGKWVTAKCVPDSIVMHIGDTLEILSNGKYKSILHRGLVNKEK
FragariaANS  241 ALTFILHNMVPGLQLFYGGKWVTAKCVPNSIVMHIGDTLEILSNGKYKSILHRGLVNKEK

BpANS        299 VRISWAVFCEPPKEKIILKPLPEVVSEEEPAIFPPRTFAQHIQHKLFRKTQDALDAK---
VitisANS     299 VRISWAVFCEPPKEKIILKPLPETVSETEPPLFPPRTFSQHIQHKLFRKTQEALLSK---
ArabANS      296 VRISWAVFCEPPKDKIVLKPLPEMVSVESPAKFPPRTFAQHIEHKLFGKEQEELVSEKND
FragariaANS  301 VRISWAVFCEPPKEKIILKPLPETVSEEEPAIFPPRTFFEHIQHKLFRQSQEALVSTKES

BpANS            -----------------------
VitisANS         -----------------------
ArabANS      356 -----------------------
FragariaANS  361 AALKSTTESALKSTKEAALISTN
```

(b)

```
BpDFR1         1 MGSEGETVCVTGAAGFIGSWLVMRLLERGYAVRATVRDPENMKKVKHLLELPKAKTHLTL
VitisDFR       1 MGSQSETVCVTGASGFIGSWLVMRLLERGYTVRATVRDPTNVKKVKHLLDLPKAETHLTL
MalusDFR       1 MGSESESVCVTGASGFIGSWLVMRLLEHGYTVRATVRDPTNQKKVKHLLDLPKAETHLTL
PopulusDFR     1 MGTEAETVCVTGASGFIGSWLIMRLLEKGYAVRATVRDPDNMKKVKHLLELPKASTHLTL
ArabDFR1       1 MVSQKETVCVTGASGFIGSWLVMRLLERGYFVRATVRDPGNLKKVQHLLDLPNAKTQLTL

BpDFR1        61 WKADLAEEGSFDEAIKGCSGVFHVATPMDFESKDPENEVIKPTINGLLGIMKACVKAKTV
VitisDFR      61 WKADLADEGSFDEAIKGCTGVFHVATPMDFESKDPGNEVIKPTIEGMLGIMKSCAAAKTV
MalusDFR      61 WKADLADEGSFDEAIQGCSGVFHVATPMDFESKDPENEVIKPTINGLLDILKACQKAKTV
PopulusDFR    61 WKADLSEEGSYDEAIQGCTGVFHVATPMDFESKDPENEVIKPTINGVLDIMRACAKSKTI
ArabDFR1      61 WKADLSEEGSYDDAINGCDGVFHVATPMDFESKDPENEVIKPTVNGMLGIMKACVKAKTV

BpDFR1       121 RRLVFTSSAGTVNVEEHQKPVYDETCWSDVEFCRTKKMTGWMYFVSKTLAEQAAWKFAKE
VitisDFR     121 RKLVFTSSAGTVNIQEHQLPVYDESCWSDMEFCRAKKMTGWMYFVSKTLAEQAAWKYAKE
MalusDFR     121 RKLVFTSSAGTVNVEEHQKPVYDESNWSDVEFCRSVKMTGWMYFVSKTLAEQAAWKYAKE
PopulusDFR   121 RKIIFTSSAGTVDVEEKRKPVYDESCWSDLDFVQGIKMTGWMYFVSKTLAEQAAWKFAED
ArabDFR1     121 RRFVFTSSAGTVNVEEHQKNVYDENDWSDLEFIMSKKMTGWMYFVSKSLAEKAAWDFAEE

BpDFR1       181 NGLDFITVIPPLVVGPFIMQSMPPSLITALSPITGNEGHYSIIKQGQFVHLDDLCNSHIF
VitisDFR     181 NNIDFISIIPTLVVGPFIMSSMPPSLITALSPITGNEAHYSIIRQGQFVHLDDLCNAHIY
MalusDFR     181 NNIDFITIIPTLVIGPFLMPSMPPSLITGLSPILRNESHYGIIKQGQYVHLDDLCLSHIY
PopulusDFR   181 NNLDFISIIPTLVVGPFIMQSMPPSLLTALSLITGNEAHYGILKQGHYVHLDDLCMSHIF
ArabDFR1     181 KGLDFISIIPTLVVGPFITTSMPPSLITALSPITRNEAHYSIIRQGQYVHLDDLCNAHIF

BpDFR1       241 LFEHPKSEGRYICSAADATIHDIGKLLREKYPEYNVPTKFKGIDEKLEIVSFSSKKVKEL
VitisDFR     241 LFENPKAEGRYICSSNDCIILDLAKMLREKYPEYNIPTEFKGVDENLKSVCFSSKKLTDL
MalusDFR     241 LYEHPKAEGRYICSSHDATIHELVKMLREKYPEYNIPTKFKGIDDNLEPVHFSSKKLREI
PopulusDFR   241 LYENPKAEGRYICNSDDANIHDLAQLLREKYPEYNVPDKFKDIDENLACVAFSSKKLTDL
ArabDFR1     241 LYEQAAAKGRYICSSHDATILTISKFLRPKYPEYNVPSTFEGVDENLKSIEFSSKKLTEM

BpDFR1       301 GFEFKYSLEDMFVGAVETCRSKGLLPPAAVENHANGKNHD--------------------
VitisDFR     301 GFEFKYSLEDMFTGAVDTCRAKGLLPPSHEKPVDGKT-----------------------
MalusDFR     301 GFEFKYSLEDMFVGAVDACRAKGLIPIPIPAEKTEAAEESNLVDVKAG------------
PopulusDFR   301 GFEFKYSLEDMFAGAVETCREKGLIPLSHKKQAVEECKENEVLPAS--------------
ArabDFR1     301 GFNFKYSLEEMFIESIETCRQKGFLPVSLSYQSISEIKVPTKNEIIEVKTGDGLTDGMKP

BpDFR1           ------------------------
VitisDFR         ------------------------
MalusDFR         ------------------------
PopulusDFR       ------------------------
ArabDFR1     361 CNKTETGVTGERTDAPMLAQQMCA
```

Fig. S3 Alignment of amino acid sequences of (a) anthocyanidin synthases of *Betula pendula*, *Vitis amurensis* (FJ645769), *Arabidopsis thaliana* LDOX (U70478) and *Fragaria* x *ananassa* (AY695817), and (b) dihydroflavonol reductases of *Betula pendula BpDFR1*, *Vitis amurensis* (FJ645768), *Malus* x *domestica* (AY227728), *Populus tremuloides* (AY147903) and *Arabidopsis thaliana* (AB033294). Identical and similar amino acids are shown with black and gray shading, respectively.

Table S1 Mean yields of internal standard (salicin, added in half of the samples extracted on a given day), used for correcting the concentrations of HPLC-quantified low-molecular weight phenolics. SE was calculated with n=2–9, depending on how many samples with internal standard added were extracted on each day.

Extraction date	Yield-%	SE
1.3.2017	71.0	1.8
2.3.2017	67.7	5.1
3.3.2017	83.8	5.5
7.3.2017	82.4	4.5
9.3.2017	68.6	4.0
13.3.2017	71.6	3.9
14.3.2017	68.0	4.2
16.3.2017	74.8	4.7
17.3.2017	72.6	2.6
20.3.2017	78.3	3.4
21.3.2017	70.7	5.8
22.3.2017	71.8	4.5
27.3.2017	77.0	3.8
29.3.2017	92.5	3.5
30.3.2017	68.1	8.3
31.3.2017	67.4	3.4
3.4.2017	73.2	0.6
5.4.2017	83.9	3.3
11.4.2017	83.1	3.5
12.4.2017	81.8	5.3
13.4.2017	75.7	3.0
18.4.2017	77.5	3.0
20.4.2017	86.4	4.4
21.4.2017	79.9	3.7
24.4.2017	82.6	3.9
4.7.2017	96.5	2.2

Table S2 Identification of low-molecular weight phenolics with UHPLC-QTOF/MS from the leaves of the control and modified *Betula pendula* lines used in the experiment, and wavelengths and standards used for quantification with HPLC-UV-DAD. Phenolic compounds were identified based on mass from UHPLC-QTOF/MS and, when this was unavailable, on UV-spectra from HPLC-UV-DAD.

		HPLC-UV-DAD				UHPLC-QTOF/MS			
	Compound	Rt, min	λ, nm	Standard	Producer	Rt, min	Theoretical mass	Measured mass	ppm
1	unidentified 1	3.3	220	salidroside	Phytolab GmbH & Co.KG., Germany				
2	dihydroflavonol 1[1]	3.6	280	ampelopsin	Extrasynthese, Genay, France				
3	gallocatechin	3.7	220	catechin	Fluka Chemie AG, Buchs, Switzerland	2.4	307.0818[6]	307.0789	-9.4
4	3,4'-dihydroxypropiophenone-3-glucoside (DPPG)	4.6	280	picein	Extrasynthese, Genay, France	2.9	327.1080[7]	327.1091	3.4
5	ampelopsin diglucoside	7.7	280	ampelopsin	Extrasynthese, Genay, France	3.2	643.1510[7]	643.1476	-5.4
6	*p*-OH-cinnamic acid monoglucoside	8.0	320	*p*-OH-cinnamic acid	Aldrich Chemie, Steinheim, Germany	3.7	349.0899[5]	349.0865	-9.9
7	flavanone 1[2]	8.4	280	eriodictyol	Extrasynthese, Genay, France				
8	catechin	9.5	220	catechin	Fluka Chemie AG, Buchs, Switzerland	3.8	291.0869[6]	291.0867	-0.6
9	ampelopsin monoglucoside	10.6	280	ampelopsin	Extrasynthese, Genay, France	4.3	505.0958[5]	505.0944	-2.8
10	coumaroylquinic acid derivative 1[3]	11.8	320	*p*-OH-cinnamic acid	Aldrich Chemie, Steinheim, Germany	4.7	361.0899[5]	361.0869	-8.4

11	ampelopsin[3]	11.9	280	ampelopsin	Extrasynthese, Genay, France	4.7	321.0610[6]	321.0595	-4.8
12	coumaroylquinic acid derivative 2	12.2	320	*p*-OH-cinnamic acid	Aldrich Chemie, Steinheim, Germany	4.9	361.0899[5]	361.0890	-2.6
14	dihydroflavonol 2[1]	13.6	280	ampelopsin	Extrasynthese, Genay, France				
15	taxifolin[4] monoglucoside	14.3	280	ampelopsin	Extrasynthese, Genay, France	5.9	489.1009[5]	489.0986	-4.7
16	flavanone 2[2]	15.5	280	eriodictyol	Extrasynthese, Genay, France				
17	taxifolin[4]	15.7	280	ampelopsin	Extrasynthese, Genay, France	6.0	305.0661[6]	305.0659	-0.8
18	flavanone 3	16.1	280	eriodictyol	Extrasynthese, Genay, France				
19	myricetin 3-galactoside	17.2	320	myricetin 3-rhamnoside	Roth, Karlsruhe, Germany	6.4	503.0802[5]	503.0786	-3.1
20	myricetin 3-glucoside	17.4	320	myricetin 3-rhamnoside	Roth, Karlsruhe, Germany	6.4	503.0802[5]	503.0786	-3.1
21	dihydroflavonol 3	18.5	220	ampelopsin	Extrasynthese, Genay, France				
22	myricetin 3-arabinoside	18.6	220	myricetin 3-rhamnoside	Roth, Karlsruhe, Germany	6.9	473.0696[5]	473.0685	-2.3
23	myricetin 3-rhamnoside	19.5	320	myricetin 3-rhamnoside	Roth, Karlsruhe, Germany	7.2	487.0853[5]	487.0853	0.1
24	quercetin 3-galactoside	20.0	320	quercetin 3-galactoside	Apin Chemical, UK	7.3	487.0853[5]	487.0853	0.1
25	quercetin 3-glucoside	20.4	320	quercetin 3-galactoside	Apin Chemical, UK	7.4	487.0853[5]	487.0842	-2.2
26	methylmyricetin 3-glucoside	20.6	320	myricetin 3-rhamnoside	Roth, Karlsruhe, Germany	7.5	517.0958[5]	517.0943	-2.9

27	quercetin 3-arabinoside	21.0	320	quercetin 3-galactoside	Apin Chemical, UK	7.8	457.0747[5]	457.0702	-9.8
28	quercetin 3-rhamnoside	23.2	320	quercetin 3-galactoside	Apin Chemical, UK	8.4	471.0903[5]	471.0898	-1.1
30	kaempferol 3-rhamnoside[3]	26.8	320	kaempferol 3-glucoside	Extrasynthese, Genay, France	9.8	455.0954[5]	455.0949	-1.1
31	flavone 1	33.4	320	apigenin	Roth Karlsruhe, Germany				
32	flavone 2	42.2	320	apigenin	Roth Karlsruhe, Germany				

Rt, retention time; λ, wavelength of quantification; ppm, mass accuracy (10^6 x (monoisotopic theoretical mass − observed mass)/observed mass);

[1]The unidentified dihydroflavonols 1 and 2 contained ampelopsin (321.0610, ppms between -2.6 and -1.0). Dihydroflavonol 1 was tentatively identified as triglucoside of ampelopsin (829.2050, ppm 4.263).

[2]Flavanone 1 was tentatively identified as eriodictyol diglucoside (613.1798, ppm 4.787) and flavanone 2 as eriodictyol (311.0519, ppm -4.051).

[3]Peaks of ampelopsin present in DFRi lines and coumaroylquinic acid derivative 1 in other lines (see also Table S3) overlapped in HPLC-UV-DAD chromatograms, and thus, could not be reliably separated. Similarly, peak of kaempferol 3-rhamnoside was overlapping with a phenolic acid.

[4]also known as dihydroquercetin

[5]$(M+Na)^+$; [6]$(M+H)^+$; [7]$(M-H)^-$

Table S3 Means ± 1 SE of phenolic concentrations in leaves of different RNA interference constructs of *Betula pendula* at different experimental replications. In each RNAi construct, levels of anthocyanidin reductase (ANR), anthocyanidin synthase (ANS), or dihydroflavonol reductase (DFR) were decreased by RNA interference in 3–4 plant lines.

Compound[1]	Control (mg g^{-1})		DFRi (mg g^{-1})		ANSi (mg g^{-1})		ANRi (mg g^{-1})	
p-OH cinnamic acid monoglucoside								
replication 1	0.26 ± 0.09	a	0.09 ± 0.01	a	0.24 ± 0.04	a	0.20 ± 0.03	a
replication 2	0.31 ± 0.11	b	0.11 ± 0.02	a	0.25 ± 0.05	a	0.18 ± 0.03	a
replication 3	0.25 ± 0.10	a	0.09 ± 0.02	a	0.17 ± 0.06	b	0.20 ± 0.02	a
coumaroylquinic acid derivative 1								
replication 1	0.86 ± 0.16		0.00 ± 0.00		1.04 ± 0.17		1.28 ± 0.29	
replication 2	0.82 ± 0.15		0.00 ± 0.00		0.88 ± 0.11		1.03 ± 0.12	
replication 3	0.71 ± 0.11		0.00 ± 0.00		0.82 ± 0.08		1.01 ± 0.14	
coumaroylquinic acid derivative 2								
replication 1	0.47 ± 0.08	a	0.20 ± 0.04	a	0.42 ± 0.03	a	0.40 ± 0.05	a
replication 2	0.47 ± 0.07	a	0.28 ± 0.05	b	0.44 ± 0.03	ab	0.40 ± 0.04	a
replication 3	0.47 ± 0.07	a	0.24 ± 0.03	c	0.47 ± 0.05	b	0.43 ± 0.05	a
*flavanone 1**								
replication 1	0.00 ± 0.00		0.06 ± 0.03	a	0.00 ± 0.00		0.00 ± 0.00	
replication 2	0.00 ± 0.00		0.20 ± 0.07	b	0.00 ± 0.00		0.00 ± 0.00	
replication 3	0.00 ± 0.00		0.37 ± 0.12	c	0.00 ± 0.00		0.01 ± 0.01	
*flavanone 2**								
replication 1	0.00 ± 0.00		0.09 ± 0.04	a	0.00 ± 0.00		0.00 ± 0.00	
replication 2	0.00 ± 0.00		0.14 ± 0.05	b	0.00 ± 0.00		0.00 ± 0.00	
replication 3	0.00 ± 0.00		0.25 ± 0.06	c	0.00 ± 0.00		0.00 ± 0.00	
*flavanone 3**								
replication 1	0.00 ± 0.00		0.06 ± 0.03	a	0.00 ± 0.00		0.06 ± 0.08	
replication 2	0.00 ± 0.00		0.04 ± 0.02	a	0.00 ± 0.00		0.01 ± 0.01	
replication 3	0.00 ± 0.00		0.09 ± 0.04	b	0.00 ± 0.00		0.01 ± 0.02	
flavone 1								
replication 1	0.09 ± 0.03		0.11 ± 0.03		0.10 ± 0.02		0.12 ± 0.03	
replication 2	0.08 ± 0.02		0.09 ± 0.02		0.08 ± 0.01		0.08 ± 0.01	
replication 3	0.06 ± 0.01		0.07 ± 0.02		0.07 ± 0.01		0.07 ± 0.02	
flavone 2								
replication 1	0.40 ± 0.08	a	1.20 ± 0.29	a	0.37 ± 0.06	a	0.52 ± 0.08	a
replication 2	0.33 ± 0.08	b	0.73 ± 0.12	b	0.30 ± 0.04	ab	0.35 ± 0.03	b
replication 3	0.31 ± 0.08	b	0.53 ± 0.13	c	0.30 ± 0.03	b	0.47 ± 0.10	a
dihydroflavonol								

*1**								
replication 1	0.00 ± 0.00		0.34 ± 0.16	a	0.00 ± 0.00		0.00 ± 0.00	
replication 2	0.00 ± 0.00		0.75 ± 0.20	b	0.00 ± 0.00		0.00 ± 0.00	
replication 3	0.00 ± 0.00		1.06 ± 0.19	c	0.00 ± 0.00		0.00 ± 0.00	
*ampelopsin diglucoside**								
replication 1	0.00 ± 0.00		0.10 ± 0.05	a	0.00 ± 0.00		0.00 ± 0.00	
replication 2	0.00 ± 0.00		0.35 ± 0.12	b	0.00 ± 0.01		0.00 ± 0.00	
replication 3	0.00 ± 0.00		0.44 ± 0.10	c	0.00 ± 0.00		0.00 ± 0.00	
ampelopsin monoglucoside								
replication 1	0.00 ± 0.01	a	1.32 ± 0.30	a	0.01 ± 0.01	a	0.06 ± 0.03	a
replication 2	0.02 ± 0.02	b	2.53 ± 0.65	b	0.00 ± 0.00	a	0.10 ± 0.02	b
replication 3	0.02 ± 0.03	b	3.54 ± 0.59	c	0.01 ± 0.01	a	0.08 ± 0.03	ab
*ampelopsin**								
replication 1	0.00 ± 0.00		10.39 ± 2.95	a	0.00 ± 0.00		0.00 ± 0.00	
replication 2	0.00 ± 0.00		20.20 ± 3.97	b	0.00 ± 0.00		0.00 ± 0.00	
replication 3	0.00 ± 0.00		26.41 ± 4.03	c	0.00 ± 0.00		0.00 ± 0.00	
*dihydroflavonol 2**								
replication 1	0.00 ± 0.00		0.72 ± 0.17	a	0.00 ± 0.00		0.19 ± 0.32	
replication 2	0.00 ± 0.00		1.14 ± 0.18	b	0.00 ± 0.00		0.00 ± 0.00	
replication 3	0.00 ± 0.00		0.90 ± 0.16	a	0.00 ± 0.00		0.00 ± 0.00	
taxifolin monoglucoside								
replication 1	0.00 ± 0.00	a	1.47 ± 0.28	a	0.02 ± 0.01	a	0.00 ± 0.01	a
replication 2	0.01 ± 0.02	a	2.32 ± 0.51	b	0.02 ± 0.02	a	0.02 ± 0.02	a
replication 3	0.00 ± 0.01	a	3.12 ± 0.45	c	0.03 ± 0.02	a	0.02 ± 0.02	a
*taxifolin**								
replication 1	0.00 ± 0.00		0.18 ± 0.05	a	0.00 ± 0.00		0.00 ± 0.00	
replication 2	0.00 ± 0.00		0.25 ± 0.07	b	0.00 ± 0.00		0.00 ± 0.00	
replication 3	0.00 ± 0.00		0.25 ± 0.04	b	0.00 ± 0.00		0.00 ± 0.00	
dihydroflavonol 3								
replication 1	0.01 ± 0.02	a	2.31 ± 0.75	a	0.01 ± 0.01	a	0.00 ± 0.00	a
replication 2	0.01 ± 0.02	a	1.63 ± 0.62	b	0.01 ± 0.01	a	0.00 ± 0.01	a
replication 3	0.01 ± 0.01	a	0.96 ± 0.37	c	0.00 ± 0.00	a	0.00 ± 0.01	a
myricetin 3-galactoside								
replication 1	0.41 ± 0.32	a	0.88 ± 0.22	a	0.95 ± 0.40	a	3.14 ± 1.23	a
replication 2	0.49 ± 0.30	a	0.85 ± 0.17	a	0.62 ± 0.19	b	1.98 ± 0.31	a
replication 3	0.37 ± 0.17	a	0.63 ± 0.11	a	0.49 ± 0.19	b	2.02 ± 0.45	a
myricetin 3-glucoside								
replication 1	0.33 ± 0.21	a	0.87 ± 0.18	a	0.67 ± 0.25	a	9.74 ± 1.95	a
replication 2	0.45 ± 0.28	a	1.46 ± 0.32	a	0.48 ± 0.12	ab	10.24 ± 1.44	ab

replication 3	0.30 ± 0.14	a	1.67 ± 0.26	b	0.42 ± 0.16	b	11.13 ± 2.20	b
myricetin 3-arabinoside								
replication 1	0.04 ± 0.06	a	0.00 ± 0.00	a	0.08 ± 0.03	a	0.57 ± 0.16	a
replication 2	0.05 ± 0.05	a	0.00 ± 0.00	a	0.06 ± 0.02	ab	0.45 ± 0.07	b
replication 3	0.03 ± 0.04	a	0.00 ± 0.01	a	0.03 ± 0.02	b	0.70 ± 0.22	c
myricetin 3-rhamnoside								
replication 1	5.34 ± 1.15	a	14.19 ± 2.06	a	8.35 ± 1.77	a	11.71 ± 2.96	a
replication 2	5.40 ± 2.10	a	11.82 ± 1.70	b	5.99 ± 0.78	b	9.64 ± 1.15	b
replication 3	4.50 ± 2.49	a	11.28 ± 1.70	b	5.55 ± 1.62	b	11.98 ± 3.56	a
methylmyricetin 3-glucoside								
replication 1	0.08 ± 0.08	a	0.13 ± 0.06	a	0.00 ± 0.00	a	0.65 ± 0.23	a
replication 2	0.09 ± 0.14	a	0.26 ± 0.08	b	0.00 ± 0.00	a	1.01 ± 0.27	b
replication 3	0.06 ± 0.09	a	0.29 ± 0.08	b	0.00 ± 0.01	a	0.98 ± 0.32	b
quercetin 3-galactoside								
replication 1	0.22 ± 0.17	a	0.19 ± 0.06	a	0.44 ± 0.15	a	0.67 ± 0.25	a
replication 2	0.28 ± 0.12	a	0.12 ± 0.04	a	0.40 ± 0.11	ab	0.38 ± 0.06	b
replication 3	0.23 ± 0.08	a	0.11 ± 0.04	a	0.31 ± 0.10	b	0.45 ± 0.17	b
quercetin 3-glucoside								
replication 1	0.09 ± 0.14		0.09 ± 0.04		0.27 ± 0.09		0.91 ± 0.25	
replication 2	0.12 ± 0.12		0.11 ± 0.04		0.28 ± 0.06		0.81 ± 0.15	
replication 3	0.10 ± 0.09		0.11 ± 0.04		0.22 ± 0.07		0.84 ± 0.26	
*quercetin 3-arabinoside**								
replication 1	0.00 ± 0.00		0.01 ± 0.01		0.00 ± 0.00		0.92 ± 0.25	
replication 2	0.00 ± 0.00		0.05 ± 0.03		0.00 ± 0.00		1.01 ± 0.20	
replication 3	0.00 ± 0.00		0.08 ± 0.03		0.00 ± 0.00		1.06 ± 0.28	
quercetin 3-rhamnoside								
replication 1	2.48 ± 0.69	a	5.39 ± 0.94	a	3.35 ± 0.49	a	5.71 ± 1.54	a
replication 2	2.51 ± 0.72	a	4.42 ± 0.49	b	3.39 ± 0.50	a	4.81 ± 0.73	b
replication 3	2.29 ± 0.69	a	4.56 ± 0.50	b	4.09 ± 0.59	b	5.81 ± 1.54	a
kaempferol 3-rhamnoside + phenolic acid								
replication 1	1.06 ± 0.20	a	1.06 ± 0.10	a	1.09 ± 0.11	a	1.02 ± 0.10	a
replication 2	1.27 ± 0.41	b	0.96 ± 0.14	a	1.06 ± 0.11	a	0.83 ± 0.09	b
replication 3	0.97 ± 0.32	a	0.73 ± 0.05	b	1.17 ± 0.11	a	1.00 ± 0.12	a
*gallocatechin**								
replication 1	0.00 ± 0.00		0.00 ± 0.00		0.24 ± 0.20	a	0.00 ± 0.00	
replication 2	0.00 ± 0.00		0.00 ± 0.00		0.77 ± 0.31	b	0.01 ± 0.02	
replication 3	0.00 ± 0.00		0.00 ± 0.00		1.41 ± 0.79	b	0.00 ± 0.00	

catechin								
replication 1	0.24 ± 0.12	a	0.00 ± 0.00	a	0.89 ± 0.28	a	0.31 ± 0.10	a
replication 2	0.65 ± 0.30	b	0.00 ± 0.00	a	2.30 ± 0.84	b	0.73 ± 0.17	b
replication 3	0.85 ± 0.27	c	0.00 ± 0.00	a	4.02 ± 1.93	c	0.92 ± 0.27	b
unidentified 1								
replication 1	0.40 ± 0.19	a	0.19 ± 0.10	a	0.39 ± 0.18	a	0.18 ± 0.05	a
replication 2	0.43 ± 0.19	a	0.31 ± 0.16	a	0.78 ± 0.32	b	0.19 ± 0.07	a
replication 3	0.42 ± 0.21	a	0.61 ± 0.30	b	0.41 ± 0.25	a	0.46 ± 0.19	b
DPPG								
replication 1	6.95 ± 1.50		6.84 ± 1.47		5.61 ± 0.72		5.68 ± 1.01	
replication 2	6.48 ± 1.28		6.67 ± 1.12		5.84 ± 0.76		5.73 ± 0.66	
replication 3	6.14 ± 1.33		5.62 ± 1.20		5.11 ± 0.51		5.61 ± 0.71	
condensed tannins								
replication 1	76.6 ± 16.8	a	28.2 ± 4.7	a	65.6 ± 14.1	a	57.6 ± 18.9	a
replication 2	102.0 ± 43.8	b	40.1 ± 10.8	b	71.8 ± 10.0	a	71.6 ± 13.5	b
replication 3	77.2 ± 17.0	a	42.6 ± 9.7	b	94.5 ± 25.8	b	77.1 ± 17.3	b

[1]Statistically significant differences between experimental replications within RNAi construct are indicated with different letters (*P*<0.05 from multivariate models including RNAi construct x replication interaction; compared only when RNAi construct x replication interaction was statistically significant). For compounds not present in the control line (*), the data were restricted to constructs in which the compounds were found, and differences between replications were analyzed with univariate models.

Table S4 Summary statistics from linear mixed models made for phenotypic traits and foliar water and nitrogen content in control and modified *Betula pendula* lines. RNAi construct, herbivory treatment, and their interaction (when it improved model fit at P<0.1 in conditional F-tests) were included as fixed factors.

	RNAi construct				Herbivory treatment				RNAi construct : treatment			
Plant trait	numDF	denDF	*F*	*P*	numDF	denDF	*F*	*P*	numDF	denDF	*F*	*P*
sqrt(LA)[1]	3	3.1	105.30	0.001	1	248.1	0.43	0.512	3	248.1	3.65	0.013
sqrt(leaf DW) [1]	3	3.2	59.36	0.003	1	242.2	1.59	0.209	3	242.1	2.16	0.094
SLA[1]	3	8.6	0.56	0.654								
SLW[1]	3	8.3	0.47	0.710								
leaf water content[2]	3	7.3	3.41	0.080								
leaf nitrogen content[1]	3	6.8	4.10	0.058								
sqrt(adaxial gland density)[2]	3	7.9	0.77	0.543	1	244.0	2.59	0.109	3	244.0	3.82	0.011
sqrt(abaxial gland density)[2]	3	7.6	0.68	0.591	1	244.1	0.01	0.943	3	244.1	2.72	0.045
Log_e(stem biomass)[2]	3	7.2	108.38	0.000								
Log_e(leaf biomass)[2]	3	6.7	85.38	0.000								
Log_e(aboveground biomass)[1]	3	5.0	24.25	0.002								

numDF, numerator degrees of freedom; denDF, denominator degrees of freedom; LA=leaf area; leaf DW=leaf dry weight; SLA=specific leaf area; SLW=specific leaf weight

[1]The models based on 159 (leaf nitrogen content) or 268 (other variables) observations of plants included random intercepts for plant lines, for each combination of experimental replication and RNAi construct, and for chambers.

[2]The models based on 268 observations of plants included random intercepts for plant line, experimental replication, and chamber.

Table S5 Summary statistics from linear mixed models testing the explaining *Epirrita autumnata* parameters based on NMS axis scores from ordination based on phenolics, nutrients and phenotypic traits of *Betula pendula* (Fig. 3b) and larval starting weight

Larval parameter[1]	NMS1[2]				NMS2[2]				larval starting weight				NMS1 : NMS2[2]			
	num DF	den DF	*F*	*P*	num DF	den DF	*F*	*P*	num DF	den DF	*F*	*P*	num DF	den DF	*F*	*P*
Log_e(consumption+10)	1	122.0	0.25	0.616	1	121.4	22.70	<0.001	1	14.7	5.36	0.036	1	120.9	0.12	0.735
Log_e(RCR+0.1)	1	122.0	0.95	0.331	1	121.3	18.77	<0.001	1	15.5	2.26	0.153	1	120.9	0.17	0.680
growth	1	116.6	0.41	0.521	1	117.0	1.86	0.175	1	88.7	20.55	<0.001	1	118.9	2.30	0.132
Log_e(RGR+1)	1	119.9	0.02	0.902	1	120.4	5.47	0.021	1	75.4	262.50	<0.001	1	121.2	2.90	0.092
Log_e(GGE+100)	1	113.7	0.46	0.499	1	113.2	6.00	0.016	1	13.4	11.15	0.005	1	114.0	0.59	0.442

numDF, numerator degrees of freedom; denDF, denominator degrees of freedom; RCR, relative consumption rate; RGR, relative growth rate; GGE, gross growth efficiency

[1]Models with 121 (GGE) or 127 (other variables) observations on late-instar *E. autumnata* included random intercepts for plant lines, experimental rounds and chambers.

[2]NMS1 and NMS2 denote axis coordinates from the ordination in Fig. 3b, based on variables in Table 3.

Methods S1 Birch EST clones used and methods for generating *Betula pendula* lines with decreased dihydroflavonol reductase (DFR), anthocyanidin synthase (ANS) or anthocyanidin reductase (ANR) activity.

The expressed sequence tag (EST) clone and the sequence amplified for generating the ANRi construct has been described before by Kosonen *et al.*, (2015). The clones Q8LP73 and Q58ZF2, putatively coding for ANS and DFR1 in *Betula pendula*, were obtained from a birch EST library (Aalto & Palva, 2006). The 1397-bp sequence of *BpANS* has a putative coding region of 1070 bp. Its amino acid sequence identity is highest with ANS proteins from *Vitis amurensis* (75%), *Fragaria x ananassa* (75%) and *Arabidopsis* (72%; Fig. **S3a**). The *BpDFR1* clone consists of 1375 bp, including a 1022-bp putative coding region, and has highest amino acid identity with DFR sequences from *Malus* x *domestica* (83%) and *Vitis amurensis* (81%; Fig. **S3b**), while its identity is 79% with *Populus tremuloides* and 77% with *Arabidopsis* DFR sequences. Sequences were aligned using CLUSTAL OMEGA (Sievers *et al.*, 2011).

Gene-specific regions were selected from each EST sequence and amplified using PCR with primers containing appropriate restriction enzyme cut sites (BamHI/ClaI and XhoI/KpnI) for cloning. The primers were 5’-AGTGGGATCCAAGCAATCCCCAAGGAGTAC-3’, 5’-GGGACTCGAGCAATCCCCAAGGAGTACGTG-3’, 5’- CCCGATCGATGAGCTCGTCGGAG -3 and 5’-ACCCGGTACCGGAGCTCGTCGGAG -3’for *BpANS* and 5’-AAGTCTCGAGGCATCGACGAGAAGTTGGAG-3’, 5’-CAAAGGATCCGACGAGAAGTTGGAGATTG-3’, 5’-GCCGATCGATTTTCAATCATGGTTCTTGCCG-3’ and 5’-ATGCGGTACCATTTTCAATCATGGTTCTTG-3’ for *BpDFR*.

PCR amplification from plasmids containing the EST clones were performed with an initial denaturation of 96°C for 2 min followed by 30 cycles of 94°C for 30 s, 52°C for 30 s and 72°C for 30 s. The PCR reactions were performed using DyNAzyme DNA polymerase (Finnzymes), and PCR products were purified using QIAquick PCR purification kits (Qiagen). After digestion with appropriate enzymes, the DNA fragments were purified using Wizard DNA Clean-UP (Promega)

and cloned into pHANNIBAL vector (Wesley *et al.*, 2001). The correct orientation of the fragments were checked by sequencing, using ABI PRISM BigDye Terminator Cycle Sequencing Kits and an ABI PRISM 310 Genetic Analyzer (PE Biosystems). The RNAi cassettes from pHANNIBAL constructs were excised by NotI and ligated into a pART27 binary vector. The constructs were inserted into the early-flowering birch clone BPM5 (Lemmetyinen *et al.*, 1998) using *Agrobacterium*-mediated transfer (Keinonen-Mettälä *et al.*, 1998).

Methods S2 Primers used for qRT-PCR analysis of the expression of *DFR*, *ANS*, and *ANR*.
The primer used for qRT-PCR analysis of ANRi lines has been described earlier Kosonen *et al.* (2015). Primers 5'-GCCTAGTCTTATTACAGCAC-3' and 5'-ATATGTGACTATTGCAGAGG-3' were used for *BpDFR1*, and 5'-AGAAGGAGAAGTACGCTAAT-3' and 5'-TAAACAAGGTGGAAGAAATA-3' for *BpANS*. The 18S ribosomal RNA gene of birch (EMBL accession number AJ279693), amplified using primers 5'-GATGCCCTTAGATGTTCTGG-3' and 5'-AATGATCTATCCCCATCAC-3', was used as an internal reference gene.

Methods S3 Conditions for rearing parental generations of *Epirrita autumnata* used in the experiment.
Wild *E. autumnata* females were sampled from Luftjokdalen in Tana located in northern Norway (70°14'53''N : 28°23'27''E) in late August 2014. The captured females (N = 350) were allowed to lay eggs on birch (*Betula pubescens*) individually in 0.3-dl plastic jars. The jars were kept in a climate chamber [photoperiod: 4 h (light) : 20 h (dark), humidity: 80 %, temperature: 12 °C] until the females died. Then, the eggs within the jars were moved outdoors under natural temperatures and light periods to induce and maintain winter diapause. In early May, the eggs were transported to a climate chamber with adequate conditions for diapause termination [photoperiod: 16 h (light) : 8 h (dark), humidity: 80 %, temperature: 5 °C]. The eggs were monitored daily. The emerging larvae (N = 2170) were reared singly on fresh *B. pubescens ad libitum* until pupation in 1.5-dl plastic jars with moist garden peat at the bottom in a climate chamber [photoperiod: 9 h (light) : 15 h (dark), humidity: 80 %, temperature: 20 °C]. After pupation, each individual was sexed based on sex-specific genital scars on the pupal cuticle,

placed back in its rearing jar, and covered with moist *Sphagnum* moss to prevent desiccation during the pupal stage in a climate chamber [photoperiod: 6 h (light) : 18 h (dark), temperature: 16 °C]. Adults emerged in September, and were mated without allowing inbreeding. Females were allowed to lay eggs singly, and the eggs were handled in a similar way as in the previous generation. Larvae used for this experiment were collected at diapause termination in spring 2016.

Methods S4 Formulating the multivariate linear mixed effect models for simultaneous testing of individual compounds as well as groups of compounds belonging to same step on the flavonoid pathway.

When multiple related chemical compounds are analyzed, it is most convenient to construct a single multivariate model for each group of related compounds, such as, for example, phenolic acids, which in this experiment includes three different compounds. The multivariate model is formulated as a special case of univariate mixed-effect model constructed by pooling all three compounds in one column of length *3n* (i.e., the first *n* elements are the first phenolic compound, the next *n* elements are the second compound and the last *n* elements the third compound). The systematic and random parts of the model are constructed using dummy variables for each of the compound, such as $\mathrm{C1}_{il}$, $\mathrm{C2}_{il}$, and $\mathrm{C3}_{il}$in the case of phenolic acids. The random part in the multivariate model included only the random effect for line. The model becomes (see, e.g., Snijders & Bosker, 1999)

$$y_{il} = \mathrm{C1}_{il}\boldsymbol{\beta}^{(1)'}\boldsymbol{x}_{il} + \mathrm{C2}_{il}\boldsymbol{\beta}^{(2)'}\boldsymbol{x}_{il} + \mathrm{C3}_{il}\boldsymbol{\beta}^{(3)'}\boldsymbol{x}_{il} + \mathrm{C1}_{il}a_i^{(1)} + \mathrm{C2}_{il}a_i^{(2)} + \mathrm{C3}_{il}a_i^{(3)} + \mathrm{C1}_{il}\varepsilon_i^{(1)} + \mathrm{C2}_{il}\varepsilon_i^{(2)} + \mathrm{C3}_{il}\varepsilon_i^{(3)} ,$$

where the parameters to be estimated are the regression coefficients for compounds 1, 2 and 3 ($\boldsymbol{\beta}^{(1)}$, $\boldsymbol{\beta}^{(2)}$ and $\boldsymbol{\beta}^{(3)}$), the variances of random line effects for each compound (var($a_i^{(1)}$), var($a_i^{(2)}$), and var($a_i^{(3)}$)), the correlations between the random effects (cor($a_i^{(1)}$, $a_i^{(2)}$), cor($a_i^{(1)}$, $a_i^{(3)}$), and cor($a_i^{(2)}$, $a_i^{(3)}$)), the residual errors for each compound (var($\varepsilon_{il}^{(1)}$), var($\varepsilon_{il}^{(2)}$), and var($\varepsilon_{il}^{(3)}$)), and the correlations between the residual errors (cor($\varepsilon_{il}^{(1)}$, $\varepsilon_{il}^{(2)}$), cor($\varepsilon_{il}^{(1)}$, $\varepsilon_{il}^{(3)}$), and cor($\varepsilon_{il}^{(2)}$, $\varepsilon_{il}^{(3)}$)). The interpretation of the fixed effects and variances of residual errors and

random effects are similar to those in the univariate models. The main benefits of such multivariate models are that (1) the estimates of cross-model correlations of random effect and residual errors tell about the joint behavior of the compounds both at line and individual plant level, and (2) they provide the possibility to do hypothesis testing for the whole group (e.g., whether the treatment has effect on some of the compounds belonging to the specified group) using standard Wald's F-tests.

References

Aalto MK, Palva ET. **2006**. Control of growth and cold acclimation in silver birch. In: Chen T, Uemura M, Fujikama S, eds. Cold hardiness in plants: Molecular genetics, cell biology and physiology. Oxfordshire, UK: CABI Publishing, 153–166.

Keinonen-Mettälä K, Pappinen A, Von Weissenberg K. **1998**. Comparisons of the efficiency of some promoters in silver birch (*Betula pendula*). *Plant Cell Reports* **17**: 356–361.

Kosonen M, Lännenpää M, Ratilainen M, Kontunen-Soppela S, Julkunen-Tiitto R. **2015**. Decreased anthocyanidin reductase expression strongly decreases silver birch (*Betula pendula*) growth and alters accumulation of phenolics. *Physiologia Plantarum* **155**: 384–399.

Lemmetyinen J, Keinonen-Mettälä K, Lännenpää M, Von Weissenberg K, Sopanen T. **1998**. Activity of the CaMV 35S promoter in various parts of transgenic early flowering birch clones. *Plant Cell Reports* **18**: 243–248.

Sievers F, Wilm A, Dineen D, Gibson TJ, Karplus K, Li W, Lopez R, McWilliam H, Remmert M, Söding J, *et al.* 2011. Fast, scalable generation of high-quality protein multiple sequence alignments using Clustal Omega. *Molecular Systems Biology* **7**.

Snijders, TAB, Bosker, RJ. 1999. *Multilevel analysis. An introduction to basic and advanced multilevel modeling.* London: Sage Publications.

Wesley SV, Helliwell CA, Smith NA, Wang M, Rouse DT, Liu Q, Gooding PS, Singh SP, Abbott D, Stoutjesdijk PA, *et al.* 2001. Construct design for efficient, effective and high-throughput gene silencing in plants. *The Plant Journal* **27**: 581–590.
